# Automated algorithm aided capacity and confidence boost in surgical decision-making training for inferior clivus

**DOI:** 10.3389/fsurg.2024.1375861

**Published:** 2024-04-18

**Authors:** Ke Tang, Bo Bu, Hongcheng Tian, Yang Li, Xingwang Jiang, Zenghui Qian, Yiqiang Zhou

**Affiliations:** ^1^ Department of Neurosurgery, Chinese PLA General Hospital, Beijing, China; ^2^ Department of Information, Medical Supplies Center of PLA General Hospital, Beijing, China; ^3^ Department of Oral and Maxillofacial Surgery, Peking University Hospital of Stomatology, Beijing, China; ^4^ Department of Otolaryngology Head and Neck Surgery, Chinese PLA General Hospital, Beijing, China; ^5^ Department of Neurosurgery, Beijing Tiantan Hospital, Capital Medical University, Beijing, China; ^6^ Department of Neurosurgery, Xuanwu Hospital, Capital Medical University, Beijing, China

**Keywords:** surgical planning, high-risk, surgical simulation, automated decision-making, confidence, risk-benefit assessment

## Abstract

**Objective:**

To assess the impact of automated algorithms on the trainees’ decision-making capacity and confidence for individualized surgical planning.

**Methods:**

At Chinese PLA General Hospital, trainees were enrolled to undergo decision-making capacity and confidence training through three alternative visual tasks of the inferior clivus model formed from an automated algorithm and given consecutively in three exemplars. The rationale of automated decision-making was used to instruct each trainee.

**Results:**

Following automated decision-making calculation in 50 skull base models, we screened out three optimal plans, infra-tubercle approach (ITA), trans-tubercle approach (TTA), and supra-tubercle approach (STA) for 41 (82.00%), 8 (16.00%), and 1 (2.00%) subject, respectively. From September 1, 2023, through November 17, 2023, 62 trainees (median age [range]: 27 [26–28]; 28 [45.16%] female; 25 [40.32%] neurosurgeons) made a decision among the three plans for the three typical models (ITA, TTA, and STA exemplars). The confidence ratings had fine test-retest reliability (Spearman's rho: 0.979; 95% CI: 0.970 to 0.988) and criterion validity with time spent (Spearman's rho: −0.954; 95%CI: −0.963 to −0.945). Following instruction of automated decision-making, time spent (initial test: 24.02 vs. 7.13 in ITA; 30.24 vs. 7.06 in TTA; 34.21 vs. 12.82 in STA) and total hits (initial test: 30 vs. 16 in ITA; 37 vs. 17 in TTA; 42 vs. 28 in STA) reduced significantly; confidence ratings (initial test: 2 vs. 4 in ITA; 2 vs. 4 in TTA; 1 vs. 3 in STA) increased correspondingly. Statistically significant differences (*P* < 0.05) were observed for the above comparisons.

**Conclusions:**

The education tool generated by automated decision-making considers surgical freedom and injury risk for the individualized risk-benefit assessment, which may provide explicit information to increase trainees’ decision-making capacity and confidence.

## Introduction

Surgeons are constantly faced with decision-making that requires assigning and comparing the values of different options or actions. Maximum surgical exposure with minimum injury risk has raised questions about the decision-making in inferior clivus surgery (1). To reach the inferior clivus, a narrow cleft between the medulla oblongata medially and the occipital condyle may cause respiratory or circulatory risk and instability of the atlantooccipital joint (2). The vertebrobasilar artery and its branches may also cause injury risk in the surgical corridor (3). Certainly, the morphological description is essential to understand the risks and benefits of the various operative steps. The operation can be guided by anatomical landmarks such as the skull base bone and exit points of cranial nerves (4). However, surgical spaces divided exquisitely by numerous permutations and combinations of the respective landmarks are often hard to follow.

Firstly, to expose the inferior clivus, the occipital condyle has been an essential landmark in categorizing the far lateral approach into transcondylar, supracondylar, and paracondylar exposures (5, 6). Which one should be chosen? Secondly, how to select a corridor close to the hypoglossal canal or jugular foramen promptly and properly? Thirdly, how do varieties of maneuverable orientations influence surgical exposure? A reasonable estimate for the operative decision-making may depend on solid empirical data. It means that in inferior clivus surgery, an inherent contradiction between a sufficient number of training procedures and never jeopardizing the safety of patients is particularly prominent. Fortunately, the simulator helped train learners before trying to operate directly on patients in the operating room.

Surgical training using a simulator is based on technical skills and knowledge of the procedure (7, 8). For example, virtual reality training has been used to increase instrument handling skills in laparoscopic surgery (9–11). However, these studies were more visible on the technical side than in the knowledge of procedural skills. Therefore, it is also necessary to provide training in non-technical skills. Decision-making may be considered an essential non-technical skill of inferior clivus surgery.

Since correct perioperative decision-making must often be rapid and under pressure, confidence may become an intrinsically driven factor for competence (12). Studies have demonstrated that surgery-based simulation experiences effectively develop and improve surgical confidence and competence (13). Some studies involved coaching nontechnical skills such as judgment and decision-making (14, 15). However, drivers of a confidence boost in decision-making training are still less well understood. Besides, automated decision-making algorithms have been developed to single out the shortest path planning from the transportation network (16). We considered that the algorithm might automate decision-making to minimize injury and maximize freedom. Therefore, we propose introducing a mathematical path planning model of the inferior clivus surgery based on an automated algorithm and exploring the impact on trainees’ decision-making capacity and confidence during training.

## Methods

### Model for surgical decision-making

We first established 50 three-dimensional (3D) skull base models and used each model to generate several surgical plans to simulate the confusion faced by the operator's mind (Supplementary Method S1 and Figures S1–S3). We used data from magnetic resonance imaging (MRI) and computed tomography (CT) acquired during Gamma Knife surgery to visualize the skull base in three-dimensional (3D) software (Mimics, Materialise US, Plymouth, Michigan). The institutional ethics committee has approved the protocol. Our previous publication describes the acquisition details of images and protocol for visualizing bony and neurovascular tissues (17, 18). Briefly, we used data from MRI before frame fixation and stereotactic CT following frame fixation of 25 patients with trigeminal neuralgia. The MRI protocols included the pre-/post-gadolinium T1 sequence, T2 SPACE sequence, and time-of-flight (TOF) sequence. In addition, we performed rigid registration to align all images and delineated anatomical structures of the posterior cranial fossa. The reconstructed images of osseous structures were obtained from CT images. Reconstructed images of the brain stem, cerebellar, and CNs were obtained from MRI T1 and T2 SPACE sequence images without contrast. Reconstructed images of the arterial system were procured from TOF images. The TOF images were then subtracted from contrast-enhanced T1 sequence images to obtain reconstructed images of the venous system. Thus, we constructed 50 skull base models as subjects (50 sides of 25 patients). Then, we used the tissue volume and risk coefficient product to quantify the injury risk and the volume of operative space to quantify the surgical freedom (Supplementary Table S1). The weight was calculated by subtracting the surgical freedom from the injury risk (Supplementary Tables S2, S3). Next, we performed Dijkstra's calculation, traversing the nodes and the paths from a starting to an endpoint and using the weight to screen the optimal plan queue automatically with the minimum sum of weights for each model (Supplementary Table S4). From the 50 models, the top three high-probability optimal plans were chosen to form three three-alternative visual tasks.

Consequently, there were three plans for each task, one optimal. Each task had its exemplar corresponding to an optimal plan. In total, three three-alternative visual tasks across the three exemplars were used as tools for surgical decision-making training.

### Setting and participants

This study took place at Chinese PLA General Hospital and received an exemption for ethical review from the institutional ethics committee. We solicited volunteers from three neurosurgical and two otolaryngological departments in the Beijing region of China through email or WeChat announcements from September 1, 2023, through November 17, 2023. The volunteers were second-year neurosurgical or otolaryngological trainees who had completed 1 clinical year of their residencies. Trainees who voluntarily participated in the study signed informed consent and initiated the three-alternative visual task.

### Decision-making training

We asked trainees to view the 3D rendering of anatomical tissues and paths of each surgical plan by modulating the transparency of each item concerned (anatomical structure or surgical corridor) and rotating the model (Supplementary Figure S4). Subsequently, an optimal and a suboptimal plan should be chosen from the three kinds of plans as the best and the next-best option to expose the inferior clivus. The selection was based on the goals that achieved maximum surgical freedom and minimum injury risk, with details shown in Table 1. The training sessions 1–3 were completed sequentially for the three exemplars on the first day of the training. The training sessions 4–6 replicated the sessions 1–3 on the second day. Then, we provided instruction by informing them about the processes and outputs of Dijkstra's automated decision-making (Supplementary Method S2). Following the instructions, training sessions 7–9 were completed for the three exemplars on the third day. Finally, the training sessions 10–12 replicates of the sessions 7–9 on the fourth day.

**Table 1:** Elements required to achieve maximum surgical freedom and minimum injury risk.

Items concerned	Structures involved	Consideration in surgical planning
Space	Paths of the step-wise exposure	Sufficient space may lead to better surgical maneuverability.
Bone	Occipital condyle, jugular tubercle, foramen magnum	It may impede sufficient exposure. Bony drilling may be time-consuming or disturb the stabilization of the occipitoatlantal joint.
Cerebellum	Cerebellar biventral lobule, cerebellar tonsils	It may impede sufficient exposure. The degree of brain retraction should be minor whenever possible.
Brainstem	Medulla oblongata	It may impede sufficient exposure. Retraction may cause respiratory or circulatory risk.
Artery	Vertebrobasilar arteries, PICA, AICA	It may impede sufficient exposure. Mechanical irritation caused by retraction may result in brain ischemia or infarction. Damage results in troublesome bleeding.
Vein	Sigmoid sinus, jugular bulb, inferior petrosal sinus	It may impede sufficient exposure. Damage results in troublesome bleeding. Ligation may cause serious complications.
Cranial nerve	CNs VI-XII	It may impede sufficient exposure. Damage results in complications such as diplopia, facial paralysis, hearing impairment, dysphagia, dyspepsia, hoarseness, and weakness of the neck muscles. Retraction of the lower cranial nerves may cause respiratory or circulatory risk.

AICA, anterior inferior cerebellar artery; CN, cranial nerve; PICA, posterior inferior cerebellar artery.

### Evaluation of decision capacity and confidence

For each training session, time spent making decision was calculated automatically from the time of training onset (loading of each exemplar) to the end of the decision-making. Hits and hit percentages of each concerned item and total hits of all items were saved automatically on completion of each time window (Supplementary Figure S4). The trainees reported the categories of the optimal and suboptimal plans and their decision confidence using a triangular scale (Supplementary Method S1 and Figure S5). Decision confidence was measured on a four-point Likert scale with “4”, “3”, “2”, and “1” representing very high, somewhat high, somewhat low, and very low, respectively. Satisfactory decision-making capacity was defined if the automated decision-making optimal plan in each exemplar was selected as the optimal plan by a trainee.

### Statistical analysis

We conducted a statistical analysis using the R programming language (Version 4.1.2). Using medians and interquartile ranges (IQR), measurement and grade data were described. Spearman's rho was used to quantify the associations during the analysis of test-retest reliability and criterion validity of the confidence ratings. For data comparison between training sessions and between item hit percentages in each session, where appropriate, the Kruskal-Wallis test was carried out for ANOVAs, and the Wilcoxon matched-pairs test was used for paired comparisons. In addition, for data comparisons between age, sex, and specialty groups, we performed unpaired comparisons using an unpaired Mann-Whitney U test. Frequency comparisons of counting data were made using Fisher exact test. A two-tailed *P* < .05 was considered statistically significant.

## Results

### Simulated decision-making difficulty and three-alternative visual task

Eleven landmarks were selected in each skull base model to generate seventeen windows, twelve paths, and seven plans (Supplementary Figures S1, S2). The optimal plan could be acquired by individualized surgical planning of Dijkstra's calculation rather than statistical comparisons between plans (Supplementary Table S3 and Figure S3).

**Figure 1:**
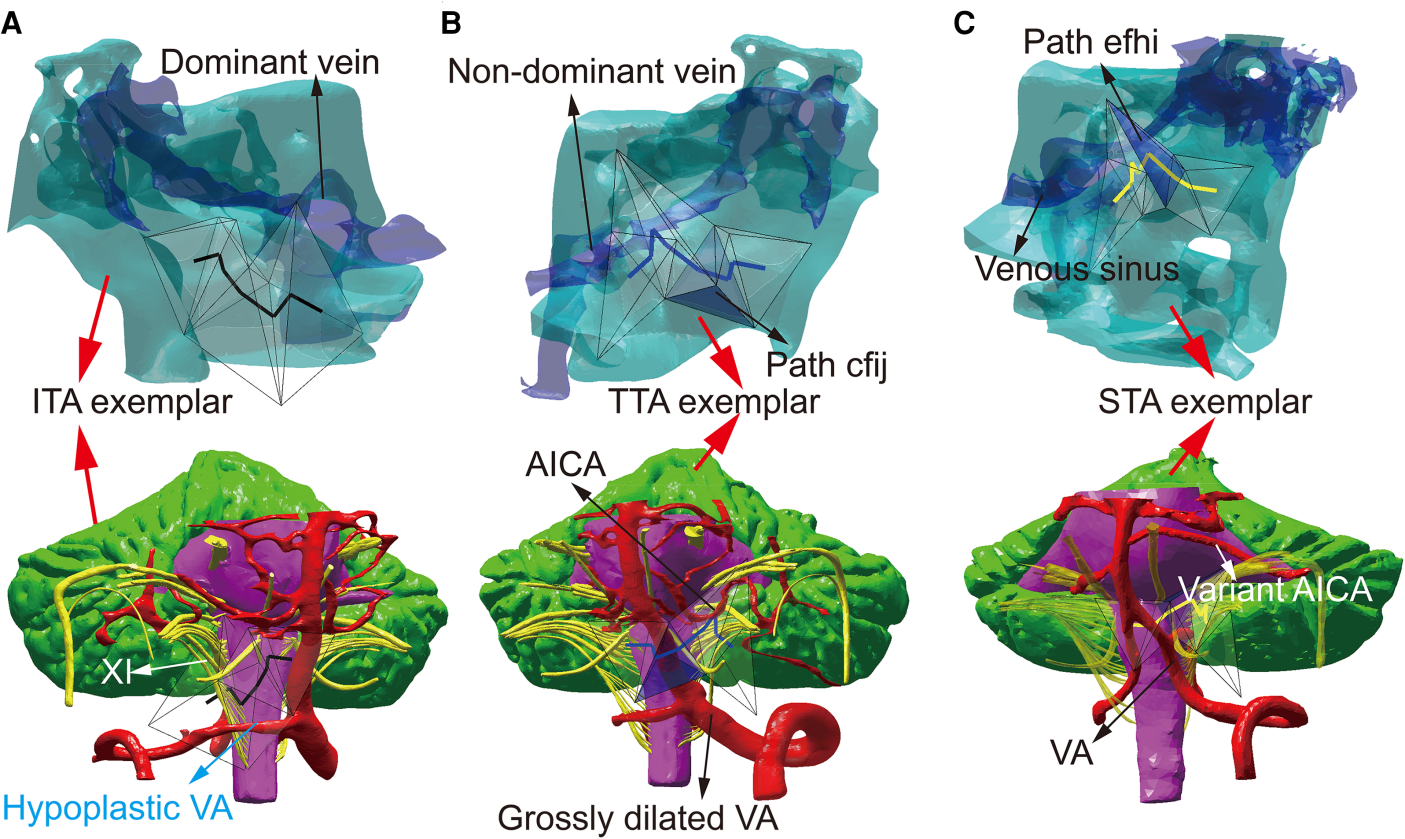
The formation of the surgical education tool. (**A**) ITA exemplar (indicated by red arrows) shows the spatial relationship between cranial base bone (cyan zone), dominant venous sinus (indicated by a black arrow), and the paths of ITA (The black lines in the transparent tetrahedrons). The paths of ITA pass through the hypoplastic VA (indicated by a blue arrow) and cranial nerve XI with a higher location (indicated by a white arrow). (**B**) TTA exemplar (indicated by red arrows) shows that the blue lines in the transparent tetrahedrons represent TTA, which exposes non-dominant venous sinus and AICA (indicated by black arrows). The paths of TTA avoid grossly dilated VA (indicated by a black arrow). The path cfij of TTA is marked as a transparent blue tetrahedron (indicated by a black arrow). (**C**) STA exemplar shows that the yellow lines in the transparent tetrahedrons represent STA. Dominant or non-dominant venous sinus and VA (indicated by black arrows) are insignificant for this case. The variant AICA (indicated by a white arrow) and the position of path efhi (shown by a transparent blue tetrahedron with a black arrow) cause lower artery volume in STA than in TTA. AICA, anterior inferior cerebellar artery; VA, vertebral artery; XI, accessory nerve.

Three plans were automatically screened out as the optimal plans and named as infra-tubercle approach (ITA), trans-tubercle approach (TTA), and supra-tubercle approach (STA) for 41 (82.00%), 8 (16.00%), and 1 (2.00%) subject, respectively (Supplementary Figure S2I). ITA, TTA, and STA surgical corridors pass under the jugular tubercle, through the jugular tubercle, and above the jugular tubercle. More details of the three approaches were described in the Supplementary Material (Supplementary Method S1). The top three high-probability plans were the just three optimal plans in all 50 subjects. Consequently, the three typical models of the above three optimal plans were named as ITA exemplar, TTA exemplar, and STA exemplar, respectively (Figure 1). Each exemplar was used to build a three-alternative visual task with three alternative plans (ITA, TTA, and STA) for trainees reporting their decisions and confidences (Supplementary Figures S6–S8).

### Volunteer recruitment and confidence ratings

Of the 64 announcements, 62 responders accepted the invitation and consented to participate, yielding a 96.88% response rate. Only 2 non-responders did not want to participate without explanation, thus perhaps leading to slight nonresponse bias. The median (IQR) age of trainees was 27 (27–28) years; 28 (45.16%) were female; 25 (40.32%) were neurosurgeons.

**Figure 2:**
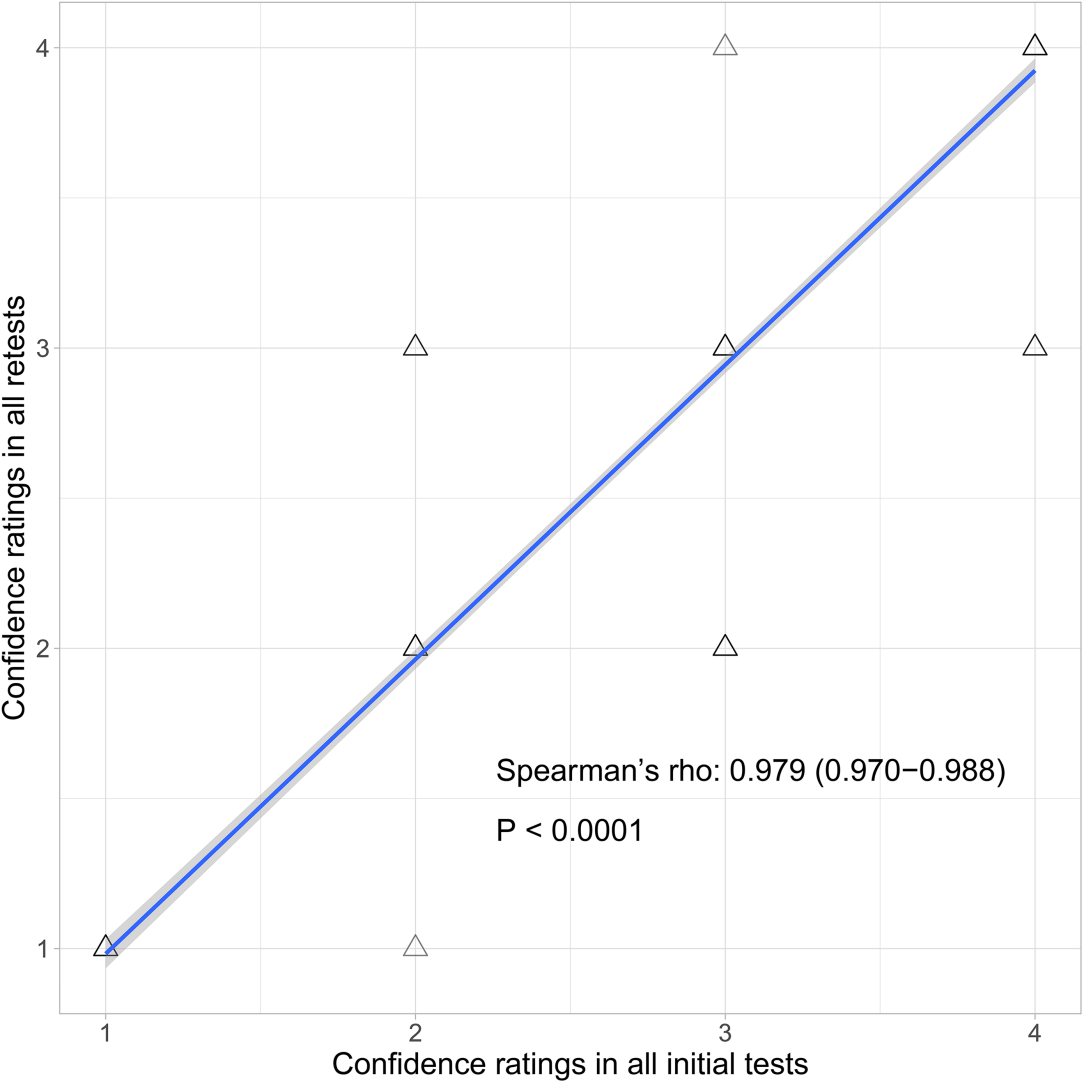
Scatter plots of test-retest reliability analysis between the confidence ratings of all initial tests (sessions 1–3 and 7–9) and all retests (sessions 4–6 and 10–12). The blue line denotes the best-fit linear correlation line. Grey shading shows 95% upper and lower confidence areas.

As Figure 2 shows, for the test-retest reliability of the confidence ratings, Spearman's rho was 0.979 [95% confidence interval (CI): 0.970–0.988]. As Supplementary Figure S9 showed, for criterion validity of the confidence ratings, Spearman's rho of association between time spent making decision and confidence ratings was −0.954 (CI: −0.963 to −0.945) in all initial tests and −0.945 (CI: −0.955 to −0.935) in all retests; and between total hits and confidence ratings was −0.917 (CI: −0.928 to −0.906) in all initial tests and −0.915 (CI: −0.926 to −0.903) in all retests.

### Decision capacity and confidence

Time spent making decision and total hits before instruction (data in sessions 1–6) were significantly more than those following instruction (data in sessions 7–12). Conversely, pre-instruction confidence ratings were significantly less than post-instruction confidence ratings (session 1 vs. session 7, session 2 vs. session 8, session 3 vs. session 9, session 4 vs. session 10, session 5 vs. session 11, session 6 vs. session 12). More details are listed in Supplementary Table S5.

Time spent making decisions in all initial tests was significantly more than in all retests (session 1 vs. session 4, session 2 vs. session 5, session 3 vs. session 6, session 7 vs. session 10, session 8 vs. session 11, session 9 vs. session 12). For total hits, comparisons failed to show significant differences between pre-instruction initial tests and pre-instruction retests in ITA and STA exemplars (session 1 vs. session 4, session 3 vs. session 6) and between all post-instruction initial tests and all post-instruction retests (session 7 vs. session 10, session 8 vs. session 11, session 9 vs. session 12). Total hits of TTA exemplar in the pre-instruction initial test were significantly more than in the pre-instruction retest (session 2 vs. session 5). For confidence ratings, comparisons failed to show significant differences between all initial tests and all retests (session 1 vs. session 4, session 2 vs. session 5, session 3 vs. session 6, session 7 vs. session 10, session 8 vs. session 11, session 9 vs. session 12).

**Figure 3:**
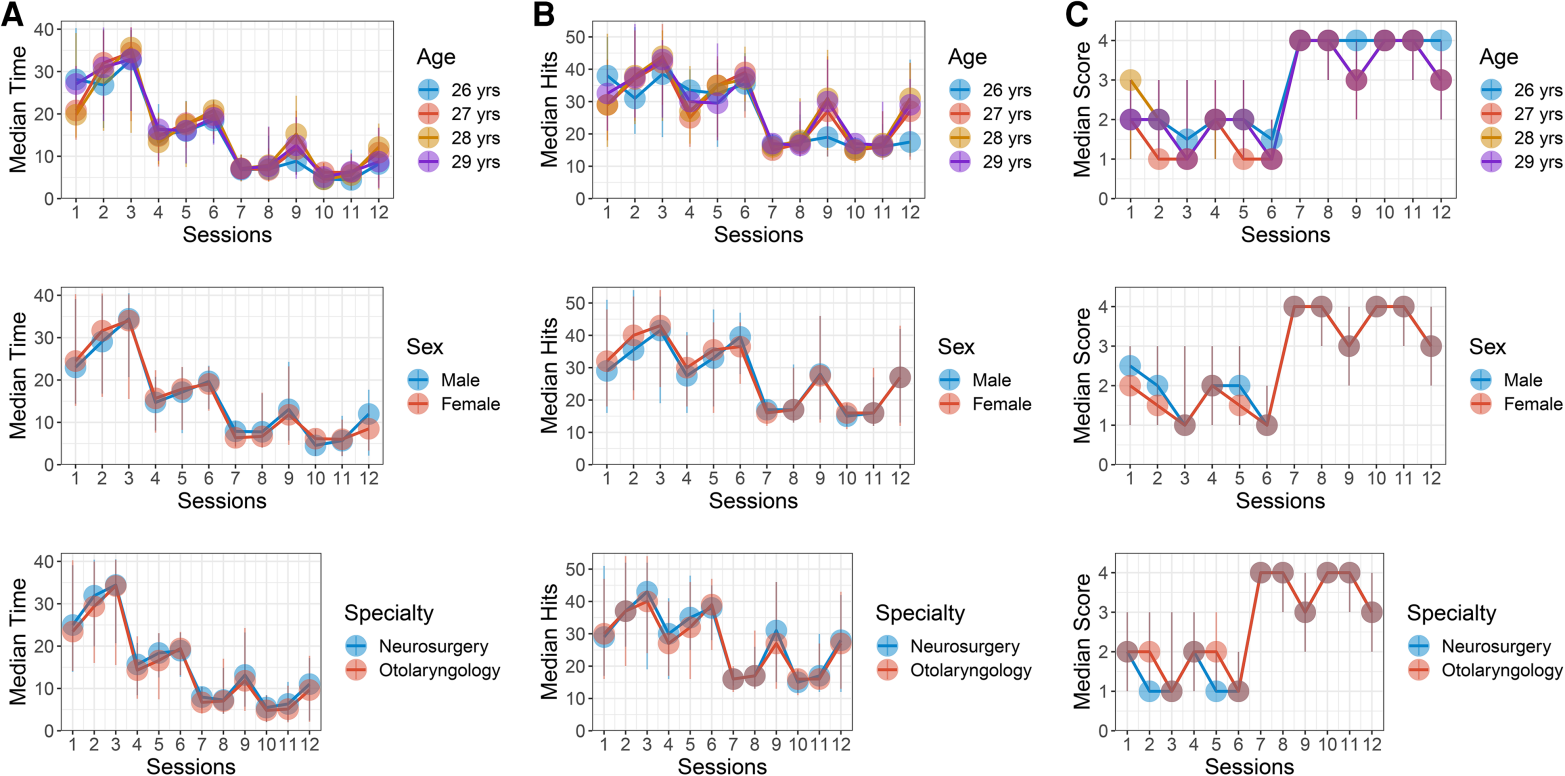
Decision capacity and confidence stratified by age, gender, and specialty subgroup in sessions 1–12. Each dot and vertical line indicate the median and range of data in each session. The dot and line color denote each subgroup. (**A**) Time spent making decision stratified by age, gender, and specialty subgroup. (**B**) Total hits stratified by age, gender, and specialty subgroup. (**C**) Confidence ratings stratified by age, gender, and specialty subgroup.

Compared to STA exemplar, ITA exemplar had less time spent making decision and total hits and higher confidence ratings (Supplementary Table S5). Apart from individual comparisons, comparisons of time spent making decision, total hits, and confidence ratings between age, sex, and specialty groups failed to show significant differences (Figure 3 and Supplementary Tables S6–S8).

### Hit percentages of concerned items

**Figure 4:**
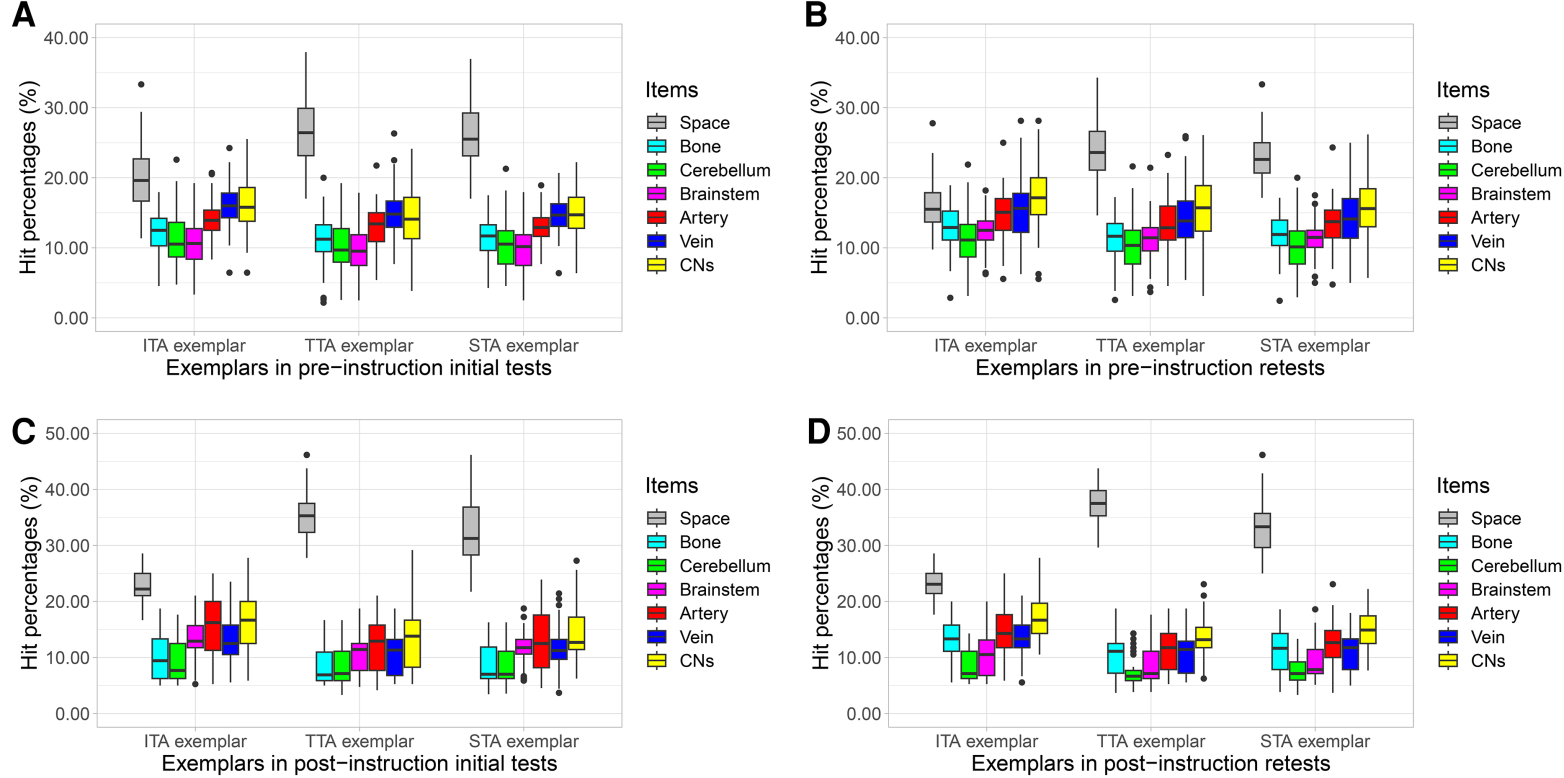
Boxplot indicates hit percentages of concerned items (surgical space, bone, cerebellum, brainstem, artery, vein, and CNs) in each session. Data are represented by a median line, a box indicating the IQR, and vertical lines representing the minimal and maximal values. Each dot represents a respondent. (**A**) Hit percentages of concerned items in pre-instruction initial tests (session 1 with ITA exemplar, session 2 with TTA exemplar, session 3 with STA exemplar). (**B**) Hit percentages of concerned items in pre-instruction retests (session 4 with ITA exemplar, session 5 with TTA exemplar, session 6 with STA exemplar). (**C**) Hit percentages of concerned items in post-instruction initial tests (session 7 with ITA exemplar, session 8 with TTA exemplar, session 9 with STA exemplar). (**D**) Hit percentages of concerned items in post-instruction retests (session 10 with ITA exemplar, session 11 with TTA exemplar, session 12 with STA exemplar). CN, cranial nerve; IQR, interquartile ranges; ITA, inferior-tubercle approach; STA, superior-tubercle approach; TTA, trans-tubercle approach.

As Figure 4 shows, operative space had the highest hit percentages; the cerebellum had the lowest. Artery, vein, and CNs also had higher hit percentages. More details are listed in Supplementary Table S9.

### Satisfactory decision-making capacity

As Supplementary Table S10 shows, each alternative plan (ITA, TTA, and STA) had four outcomes, comprising “optimal”, “suboptimal”, “select, but neither optimal nor suboptimal”, and “not select”. Before instruction, in the ITA exemplar, ITA was most likely to be selected as the optimal plan by trainees; conversely, in the STA exemplar, STA was less likely to be chosen as the optimal plan by trainees. Following instruction, the automated decision-making optimal plan in each exemplar was selected as the optimal plan by all trainees. Consequently, the number of trainees with satisfactory decision-making capacity following instruction was significantly more than before for each exemplar (Table 2).

**Table 2:** Satisfactory decision-making capacity before and after instruction.

Training sessions^a^ (*n* = 62)	Satisfactory decision-making capacity		Fisher exact test *P* value
	Pre-instruction	Post-instruction	
ITA exemplar	56 (90.32%)	62 (100.00%)	0.028
TTA exemplar	23 (37.10%)	62 (100.00%)	<0.0001
STA exemplar	3 (4.84%)	62 (100.00%)	<0.0001

ITA, inferior-tubercle approach; *n*, the number of subjects; STA, superior-tubercle approach; TTA, trans-tubercle approach.

^a^ The number of trainees with satisfactory decision-making capacity was the same in both the initial test and the retest of each exemplar.

## Discussion

Our present study may provide a new strategy for training and evaluating surgical decision confidence. In addition to neurosurgical and otolaryngologic specialties, other surgical specialties can adopt this strategy in education as long as the corresponding 3D model is developed. It is worth noting that the strategy has theoretical and practical value in mitigating concerns about patients’ rights and a long learning curve, especially in surgical education of high-risk sites. The results of our study have several implications hereinafter.

Since the operation of the inferior clivus, a representative high-risk site needs to arrive at the targeting tissue and avoid triggering danger, and a surgeon serves the simultaneous roles of expert technician and explorer. Flores's report showed that the basic far-lateral approach with partial condylectomy is sufficient for treating lesions in the region of the anterior foramen magnum (19). The surgical steps in this report were consistent with the paths in ITA of our study. Statistical comparisons between plans showed minimum volumes of the cerebellum, vein, and cranial nerve and lower weight in ITA, which implied that ITA was more likely to provide an opportunity to improve the surgical efficiency for the inferior clivus. However, decision-making based on statistical significance alone seems inadequate. The vertebral artery (VA) and medulla may pose the main limitation for ITA. TTA, with minimum volumes of the brainstem involved, may compensate for the deficiencies of ITA. This plan becomes an alternative because of the respectable surgical freedom by removing the jugular tubercle. However, excess condyle drilling and jugular bulbs may also cause postoperative complications. Little information on the above statistical significance provides insight into weighing the pros and cons of surgical decisions.

Fortunately, considering individualized anatomical traits, Dijkstra's algorithm determines the optimal plan. The core idea of the algorithm is to traverse the nodes and the paths from a starting to an endpoint and find the optimal queue with the minimum sum of weights (20). We thus considered that Dijkstra's algorithm might automate decision-making to minimize injury and maximize freedom with individualized weight calculated from the volumes of anatomical tissues and operative spaces.

Individualized surgical planning discovers that anatomical variations influence the contents of skeletal and neurovascular structures in a surgical corridor, alternating the surgical plan. As Figure 1 shows, a higher cranial nerve XI location, VA hypoplasia, and dominant sigmoid sinus may lead to screening of ITA. Vice versa, a lower cranial nerve XI location, grossly dilated VA, and non-dominant sigmoid sinus may lead to screening of TTA. Such noticeable anatomical variations can be detected easily, which may cause higher confidence ratings during the ITA exemplar training. However, the tiny difference between TTA and STA might cause decreased confidence during training of TTA and STA exemplars.

As Lisi's study showed, participants are more likely to change their minds when confidence is lower (21). Low confidence would encourage the observer to collect more information to strengthen the belief that the best option is better (22). Regarding STA exemplar, for instance, anatomical variation of the vertebrobasilar artery and its branches may also constitute a factor associated with the surgical strategy (23, 24). Yet, the variations may be subtle and imperceptible by the operator's mind directly. Therefore, in the STA exemplar, STA was less likely to be selected as the optimal plan with higher confidence before instruction. In such a situation, Dijkstra's automated decision may provide more information for trainees. If anatomic variations cause more volumes of artery branches in TTA than those in STA, the weight of STA may become less than that of TTA. Consequently, Dijkstra's algorithm screened out STA to guide trainees’ decision-making. Following the instruction of automated decision-making, the number of trainees with satisfactory decision-making capacity and higher confidence increased significantly. As Figure 4 shows, of items concerned, operative space, artery, vein, and CNs maintained higher hit percentages. Instruction of automated decision-making provided information about the above items concerned.

Questions related to “how much?” are more relevant than those related to “is it present?” For this reason, time spent making decision, total hits, and confidence ratings were employed to quantify the decision-making capacity and confidence. More time spent making decision and total hits were significantly associated with lower confidence ratings. Thus, the triangular scale of the confidence ratings had fine criterion validity. We found that time spent making decision in all retests and total hits of TTA exemplar in the pre-instruction retest were more likely to reduce, possibly due to recall. Meanwhile, the confidence ratings in all retests remained unchanged. Therefore, the fine test-retest reliability of confidence ratings might be partially affected by recall.

Whether surgeons like it or not, the time has arrived to use computers in decision-making (25). Individualized automated decision-making using computers manifested the active role of aided capacity and confidence boost. Following instruction of automated decision-making, time spent making decision and total hits reduced significantly; confidence ratings and the number of trainees with satisfactory decision-making capacity increased correspondingly.

## Limitations

It was a pilot study conducted to train the decision-making capacity and confidence. However, our 3D model cannot comprehensively represent surgical complexity, such as the impact of mass lesions and the stress tolerance mechanism for each anatomical tissue. The model was constructed from MR and CT of a patient with trigeminal neuralgia and did not consider the impact of mass lesions. Deformation and shift of the anatomical tissues caused by the mass lesions may change the ordered sequence of steps (26, 27). Thus, we need to conduct lesion-specific modeling for surgical planning. Although the study indicated that 3D models and automated decision-making instruction might increase the trainees’ decision confidence, an even finer simulation is required. Moreover, the transcondylar approaches and their variations, can be considered for both intra and extradural pathologies with different routes and techniques. The surgical manipulation of a 3D-printed model may be helpful in the future study. Increasing decision confidence may help to shorten the long learning curve to some degree; however, it is still far from effective surgeon practice. Finally, deep learning may be superior to a single algorithm in executing more advanced tasks. However, deep learning requires a large amount of training samples. The small sample size of our study may be a disadvantage. Future work will be directed toward resolving those aspects.

## Conclusion

The education tool generated by utilizing the 3D model and Dijkstra's algorithm provides a practical approach to help elucidate the concept of individualized surgical decisions based on anatomical variations. In addition, the automated decision-making instruction takes surgical freedom and injury risk into account for the risk-benefit assessment. It may provide information to increase trainees’ decision-making capacity and confidence.

## Data Availability

The datasets presented in this study can be found in online repositories. The names of the repository/repositories and accession number(s) can be found below: https://data.mendeley.com/datasets/dxn9rhh9tf/2.
