# Supplementary material for: Automated algorithm aided capacity and confidence boost in surgical decision-making training for inferior clivus
Source: Front Surg. 2024 Apr 18;11:1375861. doi: 10.3389/fsurg.2024.1375861 (PMC11063266; doi:10.3389/fsurg.2024.1375861)
Supplement: Supplementary file 1 [file Datasheet1.docx]

Supplementary Material

Automated algorithm aided capacity and confidence boost in surgical decision‐making training for inferior clivus

Ke Tang^1*†^, Bo Bu^1†^, Hongcheng Tian^2†^, Yang Li^3†^, Xingwang Jiang^4^, Zenghui Qian^5^, Yiqiang Zhou^6^

^†^These authors contributed equally to this work and share first authorship

*** Correspondence:** Ke Tang: [tangkeaccount@163.com](mailto:tangkeaccount@163.com)

# Supplementary Data

## Supplementary Method 1: Development of tools for surgical decision‐making training

**Preparation of skull base models**

We used data from magnetic resonance imaging (MRI) and computed tomography (CT) acquired during Gamma Knife surgery to visualize the skull base in three-dimensional (3D) software (Mimics, Materialise US, Plymouth, Michigan). The institutional ethics committee has approved the protocol. Our previous publication describes the acquisition details of images and protocol for visualizing bony and neurovascular tissues (1, 2). Briefly, we used data from MRI before frame fixation and stereotactic CT following frame fixation of 25 patients with trigeminal neuralgia. The MRI protocols included the pre-/post-gadolinium T1 sequence, T2 SPACE sequence, and time-of-flight (TOF) sequence. In addition, we performed rigid registration to align all images and delineated anatomical structures of the posterior cranial fossa. The reconstructed images of osseous structures were obtained from CT images. Reconstructed images of the brain stem, cerebellar, and CNs were obtained from MRI T1 sequence and T2 SPACE sequence images without contrast. Reconstructed images of the arterial system were procured from TOF images. The TOF images were then subtracted from contrast-enhanced T1 sequence images to obtain reconstructed images of the venous system. Thus, we constructed 50 models of skull base as subjects (50 sides of 25 patients).

**Simulation of confusion faced by operator's mind**

Surgical corridors of the far-lateral approach were simulated based on our previous report(3). The specific implementation steps are as follows. First, we chose the bony structure of the skull base and root entry zone (REZ) of the cranial nerve as landmarks. These landmarks are on the opposite sides of the surgical corridor, targeting the inferior clivus (Fig S1). Second, the lines between the landmarks formed triangles to simulate windows of operation entries. Third, the tetrahedrons between the windows created paths. Subsequently, we got seventeen windows and twelve paths, as Fig S1A and Fig S1B show. Finally, the windows and path combinations formed queues to generate surgical plans (Fig S1B, Fig S1C). Thus, we got seven path queues describing seven plans.

The occipital condyle has been an essential landmark in categorizing the far lateral approach into transcondylar, supracondylar, and paracondylar exposures (4, 5). In the study, as Fig S2 shows, a coronal section formed an interface at the posterior edge of the occipital condyle. The interface was categorized into △abd and △abg. The paths from △abd (paths in plans 1-4) are biased toward the caudal portion of the cranial nerves IX-XI and the hypoglossal canal medially and correspond to transcondylar and partial supracondylar exposures. In comparison, the paths from △abg (in plans 5-7) are biased toward the rostral portion of the cranial nerves IX-XI and the jugular foramen laterally and correspond to paracondylar and partial supracondylar exposures.

Since windows, paths, and queues compose a variety of operative spaces with complicated permutations and combinations, it is hard to follow and screen the operative spaces directly. Therefore, these surgical plans may reflect the confusion faced by operators.

**Automated optimal plan screen**

Volume measurement is crucial in this protocol (Fig S1D). The number of voxels of intersection between the geometry representing the path and the anatomical tissues was used to calculate the volume. (2) The surgical space and volume of anatomical tissue in each path are listed in Table S1. According to Houlihan's report (6), we used each path's volume to quantify the surgical freedom. Further, we speculated that more volume of anatomical tissues in each path might cause a higher injury risk. In addition, to indicate the difference in surgical morbidity severity caused by various tissue categories, the risk coefficients were set according to the consensus of three experienced neurosurgeons and two experienced otolaryngologists. In detail, we estimated that postoperative morbidities due to medullary and vascular injury per unit volume might be more severe. Therefore, the brainstem, artery, and vein risk coefficients were set to 1000. For comparison, the coefficient was set to 10 for bone and cerebellum due to minor trauma per unit volume. We then estimated that surgical retraction of cranial nerves to reveal the inferior clivus might cause morbidity with severity between the previous two categories. Accordingly, the coefficient was set to 500 for cranial nerves per unit volume. In the next step, we used the product of each tissue volume and risk coefficient to quantify the injury risk.

Dijkstra's algorithm has been developed to single out the shortest-path planning from the transportation network (7). We used Dijkstra's algorithm to calculate the optimal plan to serve goals that maximize surgical feasibility while minimizing the manipulation of critical anatomical tissues. The above windows were the nodes except the start node in Dijkstra's algorithm. The start node was considered an extracranial position of the operator's hands. The windows at the posterior edge of the occipital condyle formed the initial adjacent nodes relative to the start node. The weights of paths between the start node and the initial adjacent nodes were set to zero. The reason is that manipulation of the scalp, muscle, squama occipitalis, and superficial part of the cerebellar hemisphere will cause milder postoperative morbidities than brainstem and deep neurovascular structures. It also means that the path screening was begun after reaching the interface at the posterior edge of the occipital condyle (Fig S2). The remaining weight of each path anterior to the interface was calculated by the following equation (Eq):

$Weight=\left[ \left( \sum Volumes of tissues\times Risk coefficients \right)-Operative space \right]/100$ (Eq 1)

Calculating the weight by subtracting the surgical freedom from the injury risk was considered to find a minimum weight to indicate minimum injury risk and maximum surgical freedom. The weight calculated by Eq 1 for each path is listed in Table S2. The comparative results of parameters for plans 1-7 are listed in Table S3. Statistical comparisons between plans showed minimum volumes of the cerebellum, vein, and cranial nerve and lower weight in plan 1, which implied that plan 1 was more likely to provide an opportunity to improve the surgical efficiency for the inferior clivus. However, decision-making based on statistical significance alone seems inadequate. The vertebral artery (VA) and medulla may pose the main limitation for plan 1. Plan 5, with minimum volumes of the brainstem involved, may compensate for the deficiencies of plan 1. This plan becomes an alternative because of the respectable surgical freedom by removing the jugular tubercle. However, excess condyle drilling and jugular bulb may also cause injury risk. Little information on the above statistical significance provides insight into weighing the pros and cons of surgical decisions. Parallel coordinates plots of supplementary materials 3 (Fig S3A) also reflected a simulative struggle with the decision. Apart from individual sites denoting bone and brainstem, the color lines of seven plans on the parallel coordinate plots were tangled together. In the next step, Dijkstra's algorithm (Table S4) automatically found an optimal plan with a minimum sum of weights (Fig S1E). The calculation was performed in MATLAB R2020a (http://www.mathworks.com/products/matlab/).

**Three typical models and three-alternative visual tasks**

Following Dijkstra's calculation, we screened out plan 1, plan 5, and plan 6 as the optimal plans for 41 (82.00%), 8 (16.00%), and 1 (2.00%) subjects, respectively (Fig S2I). The top three high-probability plans were the three optimal plans in all 50 subjects. Parallel coordinates plots of supplementary materials 3 (Fig S3B) implied remission of the initial struggling state. The discrimination of three color lines (denoting plans 1, 5, and 6) became more distinct. Back lines (indicating plan 1) were primarily distinguished from blue and yellow lines (representing plans 5 and 6). However, blue and yellow lines were still tangled together except for sites indicating bone and surgical space. Based on the three plans, we developed tools for decision‐making training using three typical skull base models with plans 1, 5, and 6, respectively, applied as optimal decisions.

The black line in the red square frame of Fig S3A denotes one plan 1 as an optimal decision corresponding to the right side of a patient. We noted that the green and blue lines in the red square frame denoted one plan 2 and one plan 5 with a lower weight. The three color lines corresponded to the same side (right) of the same patient. Since plan 1 had been chosen as the optimal decision, plans 2 and 5 were given up by Dijkstra's calculation regardless of the lower weights. As Fig 1A shows, the right side became an exemplar model. The blue line in the blue square frame of supplementary materials 3 (Fig S3B) denotes one plan 5 as an optimal decision corresponding to the left side of the same patient mentioned above. The left side also became an exemplar model (Fig 1B). Dijkstra's calculation screened out one plan 6 as an optimal decision for an individual subject. The individual subject was the left side of a patient, which also became an exemplar model (Fig 1C). The yellow line denoting the plan 6 of the exemplar is located in the magenta square frame of Fig S3B with tangled lines (each line corresponds to a different subject).

The surgical corridor of plan 1 passes under the jugular tubercle; thus, plan 1 was named as infra-tubercle approach (ITA). The surgical corridor of plan 5 passes through the jugular tubercle; thus, plan 5 was named as trans-tubercle approach (TTA). The surgical corridor of plan 6 passes above the jugular tubercle, therefore, plan 6 was named as supra-tubercle approach (STA). Consequently, the three typical exemplar models of the above three optimal plans were named the ITA exemplar, TTA exemplar, and STA exemplar, respectively (Fig 1). Finally, the three-alternative visual task with ITA, TTA, and STA for each exemplar model was formed. The 3D rendering of each exemplar model (Fig S4) was implemented for each three-alternative visual task under the MATLAB software environment (http://www.mathworks.com/products/matlab/). At the end of the decision-making training, the trainees reported the categories of optimal and suboptimal plans and their decision confidence using the following triangular scale.

**Reporting of decision‐making and confidence**

Guided by a previous study with Li et al. (8), we designed a triangular scale for trainees to report their decision confidence. First, we denoted the triangle's three vertices as plan names in the three-alternative decision task. After this, the region between the triangle center and each vertice was divided into four segments named"4", "3", "2", and "1" at a near-to-far distance from each vertice, representing very high, somewhat high, somewhat low, and very low, respectively. Each region formed a four-point Likert scale of confidence report (Fig S5). Trainees made two responses: the category of the optimal plan and their confidence in their decision on a four-point Likert scale. In addition, we used lines between the triangle center and vertices to divide the triangle into three areas for reporting the next-best plan. Each area involves two vertices of the triangle. The next-best plan is another vertice involved in the area where the optimal plan is situated. Option "1" in the Likert scale denotes none of the optimal and suboptimal choices.

**References**

1. Z. H. Qian, X. Feng, Y. Li and K. Tang: Quantification of Surgical Route Parameters for Exposure of the Jugular Foramen Via a Trans-Mastoidal Approach Exposing Jugular Foramen in Three-Dimensional Visualization Model. *J Craniofac Surg*, 29(3), 787-791 (2018) doi:10.1097/SCS.0000000000004234

2. K. Tang, Z. H. Qian, X. Feng and Y. Li: Gridding Microsurgical Anatomy of Far Lateral Approach in the Three-Dimensional Model. *J Craniofac Surg*, 30(1), 87-90 (2019) doi:10.1097/SCS.0000000000004849

3. K. Tang, X. Feng, XiaodongYuan, Y. Li and XinyueChen: Volumetric comparative analysis of anatomy through far-lateral approach: surgical space and exposed tissues. *Chin Neurosurg J*, 8(1), 1 (2022) doi:10.1186/s41016-021-00268-8

4. H. T. Wen, A. L. Rhoton, Jr., T. Katsuta and E. de Oliveira: Microsurgical anatomy of the transcondylar, supracondylar, and paracondylar extensions of the far-lateral approach. *J Neurosurg*, 87(4), 555-85 (1997) doi:10.3171/jns.1997.87.4.0555

5. E. Shiban, E. Torok, M. Wostrack, B. Meyer and J. Lehmberg: The far-lateral approach: destruction of the condyle does not necessarily result in clinically evident craniovertebral junction instability. *J Neurosurg*, 125(1), 196-201 (2016) doi:10.3171/2015.5.JNS15176

6. L. M. Houlihan, D. Naughton and M. C. Preul: Volume of Surgical Freedom: The Most Applicable Anatomical Measurement for Surgical Assessment and 3-Dimensional Modeling. *Front Bioeng Biotechnol*, 9, 628797 (2021) doi:10.3389/fbioe.2021.628797

7. Y. Zhang, Y. Su, J. Yang, J. Ponce and H. Kong: When Dijkstra Meets Vanishing Point: A Stereo Vision Approach for Road Detection. *IEEE Trans Image Process*, 27(5), 2176-2188 (2018) doi:10.1109/TIP.2018.2792910

8. H. H. Li and W. J. Ma: Confidence reports in decision-making with multiple alternatives violate the Bayesian confidence hypothesis. *Nat Commun*, 11(1), 2004 (2020) doi:10.1038/s41467-020-15581-6

## Supplementary Method 2: Instruction about the rationales, processes, and outputs of Dijkstra's automated decision-making

The rationale of automated decision-making was explained as follows. More path volume is a favorable factor associated with more surgical feasibility. This occurs owing to adequate space and illumination permitting manipulation of instruments. Nevertheless, if a path has more volumes of anatomical structures, the congestion may be detrimental to surgical freedom. In addition, injuries related to various tissue categories have different surgical morbidity severity.

Consequently, the product of the risk coefficients and the volumes of anatomical structures can be used to quantify the surgical risk. For example, postoperative morbidities due to medullary and vascular injury per unit volume may be more severe. Therefore, the brainstem, artery, and vein risk coefficients were set to 1000. In contrast, bone and cerebellum injuries per unit volume may cause less severe morbidities. Consequently, the coefficient was set to 10 for bone and cerebellum due to minor injuries per unit volume. Surgical retraction of cranial nerves may cause morbidity with severity between the previous two categories. Accordingly, the coefficient was set to 500 for cranial nerves per unit volume. The weight of each path calculated by subtracting the surgical risk from the path volume can be used to guide decision-making for surgical planning. If a plan has a minimum sum of all path weights, the plan may be the optimal choice with maximum surgical freedom and minimum injury risk.

Next, we told trainees that an automated decision-making algorithm could automatically screen the optimal plan with minimum sum of all path weights. The anatomy of each plan is detailed below.

There were three plans, the infra-tubercle approach (ITA), trans-tubercle approach (TTA), and supra-tubercle approach (STA), screened as optimal decisions for three exemplars (Figs S6-S8). The surgical corridor of ITA started from the medial occipital hemicondyle, stepping over the hypoglossal canal. The inferior portion of the corridor encountered the anterior medullary segments of the posterior inferior cerebellar artery (PICA) below the biventral lobule of the cerebellum. Directing the corridor anteriorly and medially, the ITA corridor reached the inferior clivus below the cranial nerve VI. Compared to the 3D rendering of ITA, the lateral portion of the TTA corridor contained the medial part of the jugular foramen. Next, it exposed the superior portion of the olive. Then, anteriorly and inferiorly, the corridor passed the jugular tubercle through the cranial nerve XII. Finally, the TTA corridor exposed the inferior clivus and basal artery (BA). The surgical corridor of STA showed similarities to that of TTA. It was more remote from the vertebral artery (VA) but closer to the jugular foramen than the corridor position of ITA. Hence, the jugular bulb might cause injury risk during exposure. The corridor passed above the jugular tubercle and also passed through where the cranial nerves X-XI converged, roughly the same exposure as TTA.

Finally, the outputs of Dijkstra's automated decision-making for each exemplar were informed for each trainee. The interpretation of the screening results is detailed below.

In training session 1 and its replicate (training session 4), the exemplar was the ITA exemplar (Fig S6). The higher cranial nerve XI location, VA hypoplasia, and dominant sigmoid sinus led to the lowest weight and automated screening of ITA.

In training session 2 and its replicate (training session 5), the exemplar was the TTA exemplar (Fig S7). The grossly dilated VA shifted towards the ITA corridor. The ITA corridor involved more volume of cranial nerves IX-XI for the lower location. Thus, the ITA corridor had a higher weight. Conversely, the TTA corridor formed a better operative space and avoided the VA. Meanwhile, the STA corridor involved more volume of the premeatal segment of the AICA, which caused more weight. Therefore, The lower cranial nerve XI location, grossly dilated VA, and non-dominant sigmoid sinus of the TTA exemplar led to the lowest weight and automated screening of TTA.

In training session 3 and its replicate (training session 6), the exemplar was the STA exemplar (Fig S8). The surgical corridor of ITA was closer to the VA and medulla, which caused more weight. The TTA corridor also involved the VA. Both TTA and STA avoided the AICA. Compared to TTA, STA avoided the VA. Therefore, The anatomic variations caused more volumes of artery branches in the TTA corridor than in the STA corridor. The weight of STA becomes less than that of TTA. Consequently, Dijkstra's algorithm screened STA.

# Supplementary Figures and Tables

## Supplementary Figures


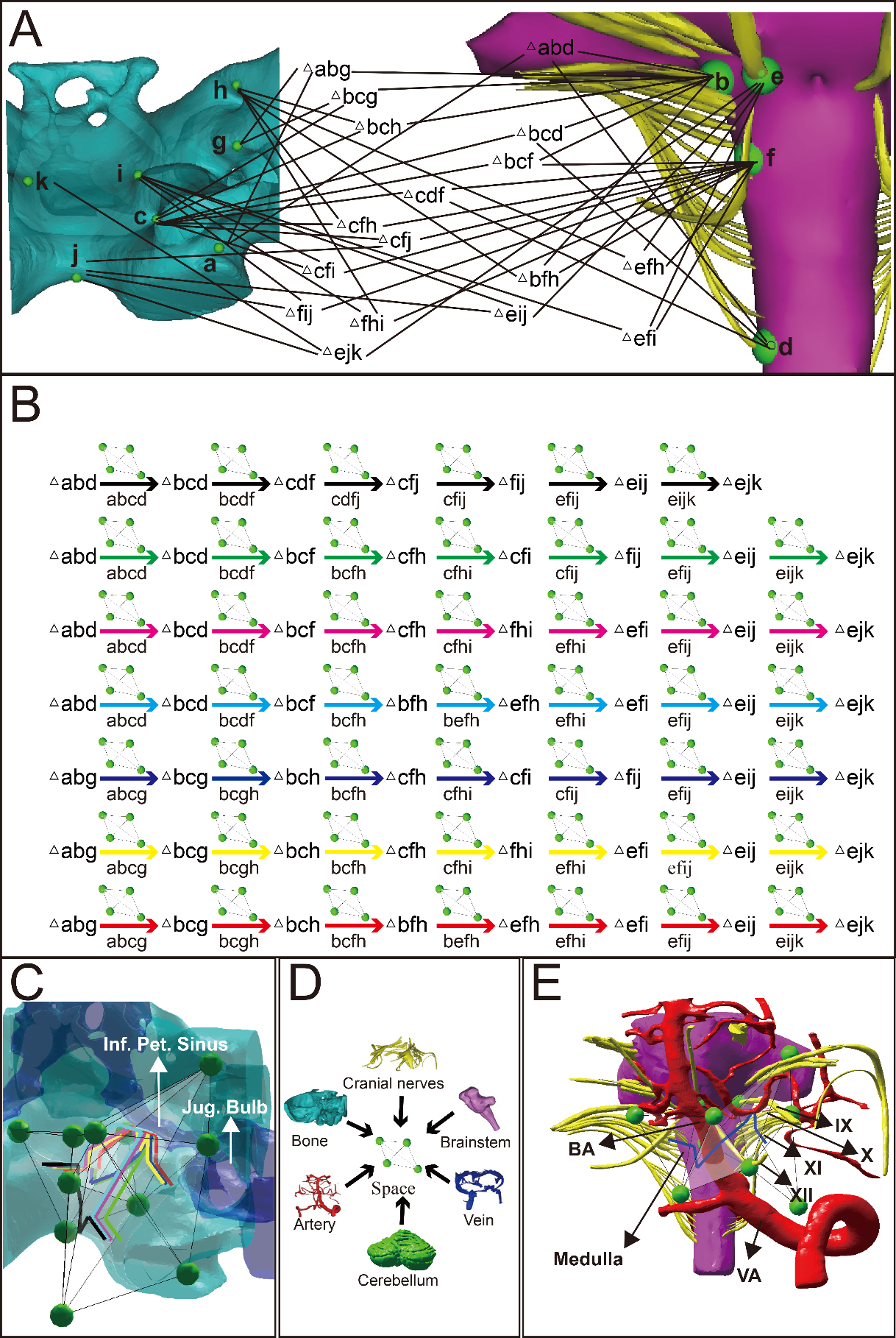


**Supplementary Figure 1.** A. In the workflows, we sequentially chose landmarks as follows: a, occipital condyle (posterior edge); b, cranial nerve IX REZ; c, hypoglossal canal; d, cranial nerve XI REZ (caudal edge); e, cranial nerve VI REZ; f, cranial nerve XII REZ; g, jugular foramen (superior edge); h, internal acoustic canal (superior edge); i, jugular tubercle (anterior edge); j, inferior clivus; and k, contralateral jugular tubercle. The seventeen windows of operation entries (△abd, △abg, △bcd, △bcf, △bcg, △bch, △bfh, △cdf, △cfh, △cfi, △cfj, △efh, △efi, △eij, △ejk, △fhi, △fij) were formed by combinations of landmarks. B. The twelve tetrahedrons (abcd, abcg, bcdf, bcgh, bcfh, cdfj, befh, cfhi, cfij, efhi, efij, eijk) between the windows were formed to simulate paths. The windows and paths comprised queues to simulate seven plans marked as arrows combined and with color legend as follows: black indicates plan 1; green, plan 2; magenta, plan 3; cyan, plan 4; blue, plan 5; yellow, plan 6; and red, plan 7. C. The plans were marked as lines with the same color legend above. The relationships of triskelions representing paths, color lines representing plans, bone (cyan zone), and veins (blue zones indicated by white arrows) are shown in a 3D view. D. Dijkstra's automated decision-making was calculated based on volumes of bone, cerebellum, brainstem, cranial nerve, artery, vein, and operative space. Black arrows indicate that each anatomical tissue involved in the individual path space has its own volume. E. For example, Dijkstra's algorithm selected the blue line in the triskelion combination representing plan 5. The 3D view shows the relationships between paths and neurovascular structures (indicated by black arrows). BA = basal artery; Inf. = inferior; Jug. = jugular; Pet. = petrosal; REZ = root entry zone; VA = vertebral artery; IX = glossopharyngeal nerve; X = vagus nerve; XI = accessory nerve; XII = hypoglossal nerve; 3D = 3-dimensional.


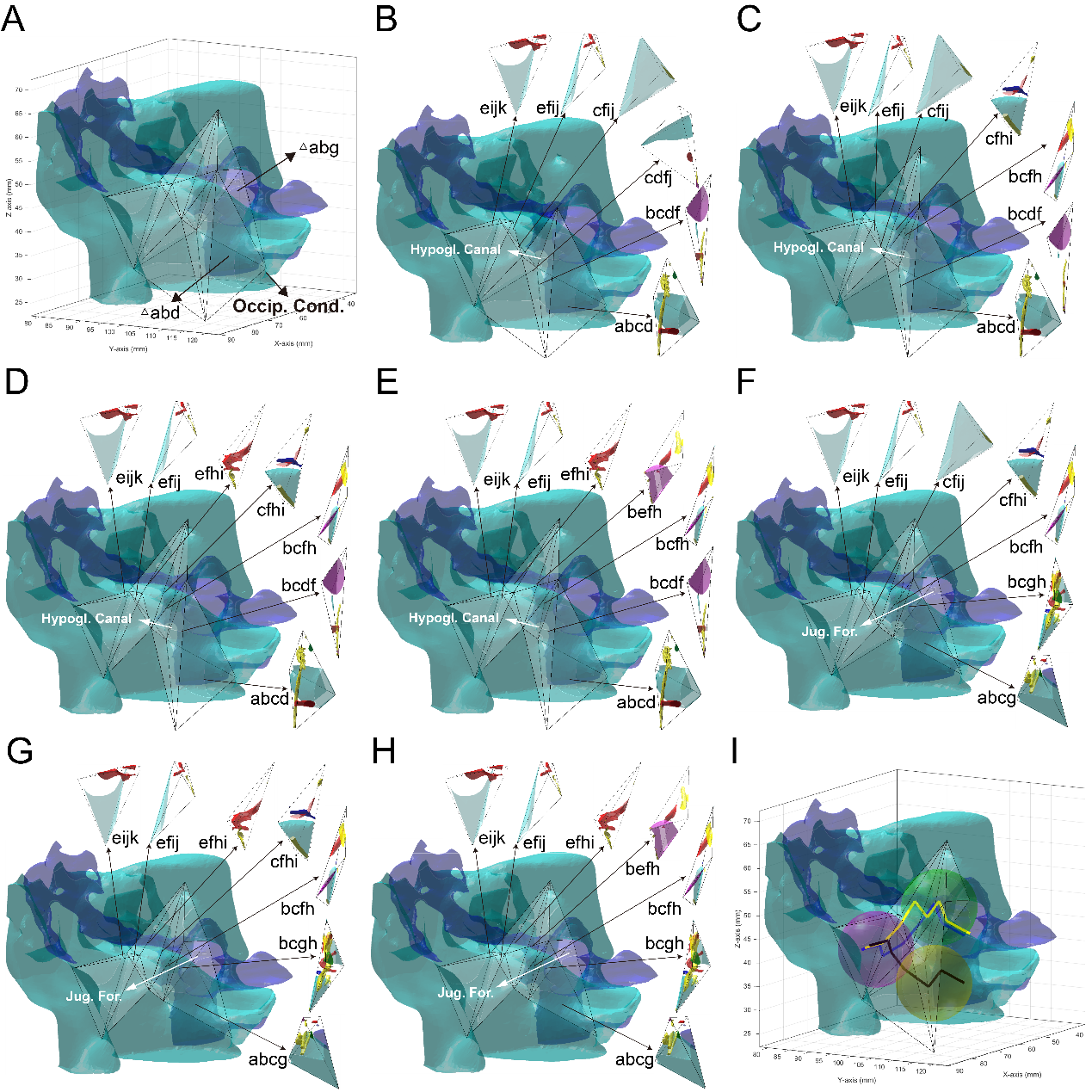


**Supplementary Figure 2.** A. A 3D view of all pending paths on the right side. The path screening was begun after reaching the interface at the posterior edge of the occipital condyle, indicated by black arrows. The interface was categorized into △abd and △abg, indicated by black arrows. B-H. A 3D view of the exposures of the paths in plans 1-7. Each tetrahedron representing a path involves anatomical structures with the color legend: cyan indicates bone; green, cerebellum; magenta, brainstem; red, artery; blue, vein; and yellow, cranial nerves. The volume of each anatomical structure can be measured. Difficult choices faced during the operation were simulated by the twelve paths abcd, abcg, bcdf, bcgh, bcfh, cdfj, befh, cfhi, cfij, efhi, efij, and eijk. Queues with various path combinations formed plans 1-7. The paths from △abd (paths in plans 1-4) are medially biased toward the hypoglossal canal (indicated by a white arrow). In comparison, the paths from △abg (in plans 5-7) are biased toward the jugular foramen (indicated by a white arrow) laterally. B. Plan 1 comprised six paths, abcd, bcdf, cdfj, cfij, efij, and eijk, indicated by black arrows sequentially. C. Plan 2 comprised seven paths, abcd, bcdf, bcfh, cfhi, cfij, efij, and eijk, indicated by black arrows sequentially. D. Plan 3 comprised seven paths: abcd, bcdf, bcfh, cfhi, efhi, efij, and eijk, indicated by black arrows sequentially. E. Plan 4 comprised seven paths, abcd, bcdf, bcfh, befh, efhi, efij, and eijk, indicated by black arrows sequentially. F. Plan 5 comprised seven paths, abcg, bcgh, bcfh, cfhi, cfij, efij, and eijk, indicated by black arrows sequentially. G. Plan 6 comprised seven paths, abcg, bcgh, bcfh, cfhi, efhi, efij, and eijk, indicated by black arrows sequentially. H. Plan 7 comprised seven paths, abcg, bcgh, bcfh, befh, efhi, efij, and eijk, indicated by black arrows sequentially. I. The screening result of Dijkstra's algorithm showed the top three high-probability plans, plan 1 (black lines), plan 5 (blue lines), and plan 6 (yellow lines) for the inferior clivus region (magenta sphere), the region near the hypoglossal canal (yellow sphere) and the region near the jugular foramen (green sphere). Plan 1 (41/50, 82.00%; by Dijkstra's screening) passes along the edge of the foramen magnum and exposes the region near the hypoglossal canal. Plans 5 (8/50, 16.00%) and 6 (1/50, 2.00%) pass through the jugular foramen and the jugular tubercle. Compared to plan 5, the path cfij was replaced by the path efhi in plan 6. Cond. = condyle; For. = foramen; Hypogl. = hypoglossal; Jug. = jugular; Occip. = occipital; 3D = three-dimensional.


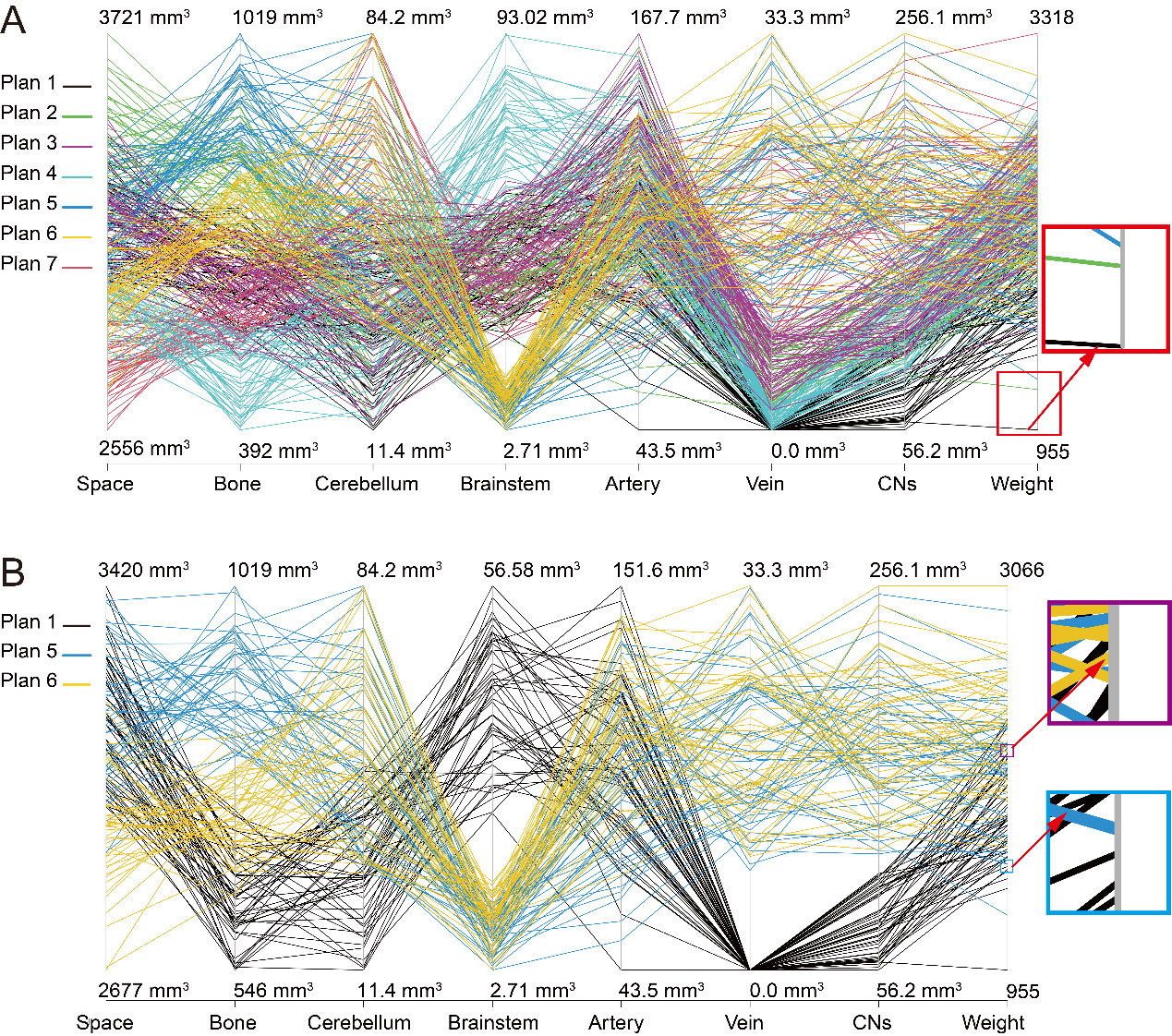


**Supplementary Figure 3.** A. The parallel coordinates plots show the lines denoting seven plans for 50 subjects (50 sides of 25 patients). The same color legend as Fig S1B applies here. Apart from individual sites denoting bone and brainstem, the color lines of seven plans on the parallel coordinates plots were tangled together. The parallel coordinates plots reflected a simulative struggle with the decision. A skull base sample of plan 1, indicated by a red arrow in the red square frame, was selected as an exemplar of the infra-tubercle approach (ITA). The exemplar was the right side of a patient and named the ITA exemplar (Fig 1A). We noted that the green and blue lines in the red square frame denoted plans 2 and 5 with a lower weight. The three color lines corresponded to the same side (right) of the same patient. Since plan 1 had been chosen as the optimal decision, plans 2 and 5 were given up by Dijkstra's calculation regardless of the lower weights. B. Following Dijkstra's calculation, we screened out plan 1, plan 5, and plan 6 as the optimal plans. Lines on the parallel coordinates plots denote plans 1 (black lines), 5 (blue lines), and 6(yellow lines). The parallel coordinates plots implied remission of the initial struggling state. The discrimination of three color lines (denoting plans 1, 5, and 6) became more distinct. Back lines (indicating plan 1) were primarily distinguished from blue and yellow lines (representing plans 5 and 6). However, blue and yellow lines were still tangled together except for sites indicating bone and surgical space. The blue line in the blue square frame denotes plan 5 as an optimal decision corresponding to the left side of the same patient mentioned above. The left side became an exemplar model of the trans-tubercle approach (TTA) and was named the TTA exemplar (Fig 1B). Dijkstra's calculation screened out plan 6 as an optimal decision for an individual subject. The yellow line denoting plan 6 of a skull base model is in the magenta square frame with tangled lines (each line corresponds to a different subject). The individual subject was the left side of a patient, which became an exemplar model of the supra-tubercle approach (STA) and named the STA exemplar (Fig 1C). CNs = cranial nerves; ITA = inferior-tubercle approach; STA = superior-tubercle approach; TTA = trans-tubercle approach.


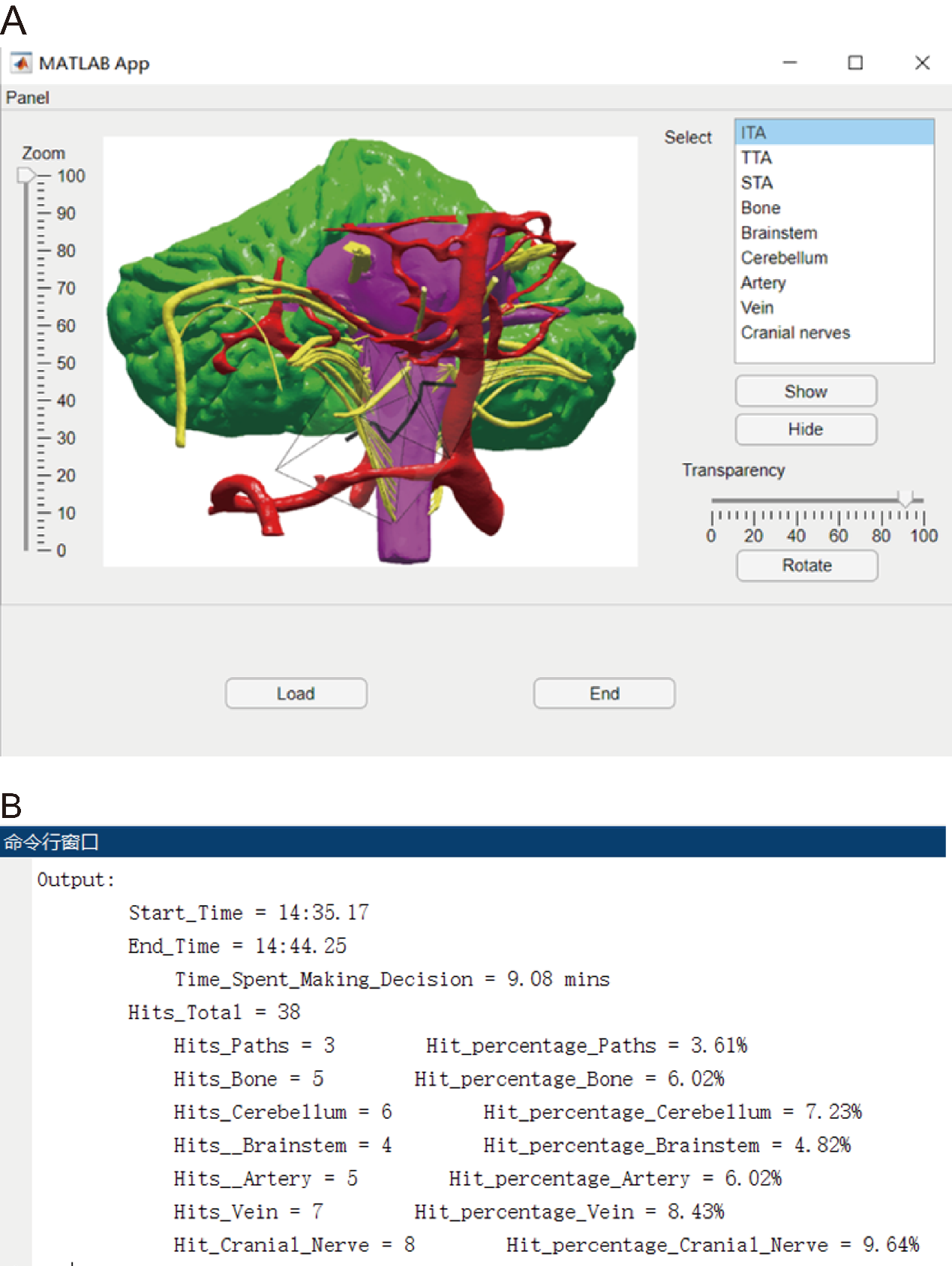


**Supplementary Figure 4.** A. A screenshot of the graphical user interface for the three-alternative visual task. The 3D rendering of anatomical tissues and paths of each surgical plan could be viewed by modulating the transparency of each item concerned (anatomical structure or surgical corridor) and rotating the model. B. A screenshot of the output of the time of training onset, the time of the end of the decision-making, the time spent making decision, the hits and hit percentage of each concerned item, and the total hits of all items on completion of a time window. ITA = inferior-tubercle approach; STA = superior-tubercle approach; TTA = trans-tubercle approach; 3D = three-dimensional.


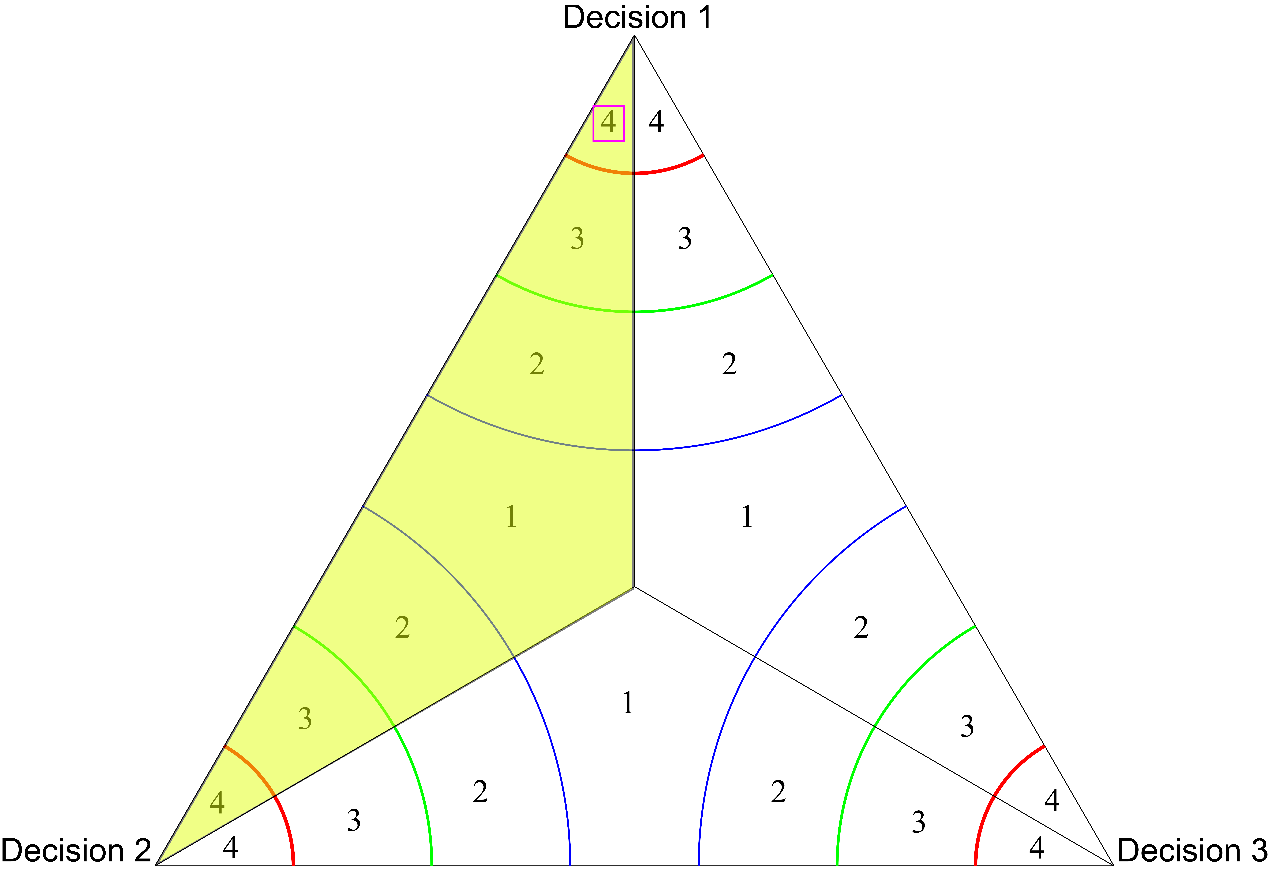


**Supplementary Figure 5.** The triangle's three vertices are denoted as decision names in the three-alternative decision task. After this, the region between the triangle center and each vertice is divided into four segments named "4", "3", "2", and "1" at a near-to-far distance from each vertice, representing very high, somewhat high, somewhat low, and very low, respectively. Each region forms a four-point Likert scale of confidence report. In the example, a trainee used a magenta square to choose "Decision 1", and the confidence in the decision was very high, denoted by "4". The yellow transparent area illustrates one of three areas divided by lines between the triangle center and vertices and involves two vertices denoting "Decision 1" and "Decision 2". Since "Decision 1" had been chosen as the optimal choice, the next-best choice was "Decision 2". Option "1" in the Likert scale denotes none of the optimal and suboptimal decisions.


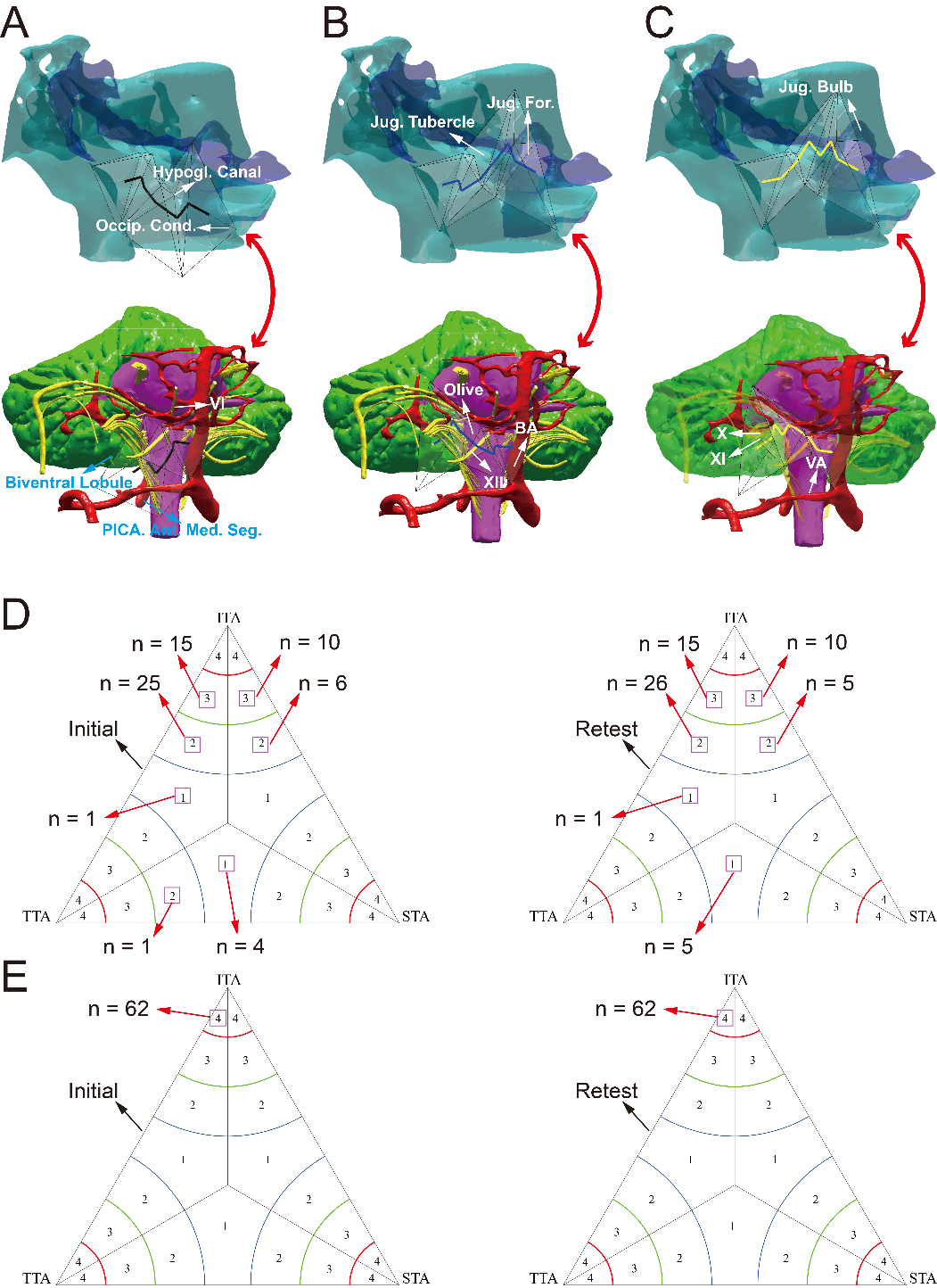


**Supplementary Figure 6.** A-C. The red two-direction arced arrow indicates two opposite sides of each surgical corridor in the ITA exemplar. A. A. The 3D rendering showed that the surgical corridor of ITA (The black lines in the transparent tetrahedrons) started from the medial occipital hemicondyle (indicated by white arrows), stepping over the hypoglossal canal (indicated by white arrows). The inferior portion of the corridor encountered the anterior medullary segments of the PICA (indicated by blue arrow) below the biventral lobule of the cerebellum (indicated by blue arrow). Directing the corridor anteriorly and medially, we reached the inferior clivus below the cranial nerve VI (indicated by a white arrow). B. Compared to the 3D rendering of ITA, the lateral portion of the TTA corridor (The blue lines in the transparent tetrahedrons) contained the medial part of the jugular foramen (indicated by a white arrow). Next, it exposed the superior portion of the olive (indicated by a white arrow). Then, anteriorly and inferiorly, the corridor passed above the jugular tubercle (indicated by a white arrow) through the cranial nerve XII (indicated by a white arrow). Finally, the corridor exposed the inferior clivus and BA (indicated by a white arrow). C. The surgical corridor of STA (The yellow lines in the transparent tetrahedrons) showed similarities to TTA. It was more remote from the VA (indicated by a white arrow); however, it was closer to the jugular foramen than the corridor position of ITA. Hence, the jugular bulb (indicated by a white arrow) might cause injury risk during exposure. The corridor also passed through where the cranial nerves X-XI (indicated by white arrows) converged, roughly the same exposure as TTA. D. Left panel. Pre-instruction initial confidence ratings (indicated by a black arrow) showed that 25 trainees (25/62, 40.32%) chose ITA as the best option, TTA as the next-best option with 2 points, and 15 trainees (15/62, 24.19%) with 3 points; 1 trainee (1/62, 1.61%) could not make decision between ITA and TTA with 1 point; and 6 trainees (6/62, 9.68%) chose STA as the next-best option with 2 points, and 10 trainees (10/62, 16.13%) with 3 points; and 1 trainee (1/62, 1.61%) chose TTA as the best option, STA as the next-best option with 2 points; and 4 trainees (4/62, 6.45%) could not make decision between TTA and STA with 1 point. Consequently, in total, 5 trainees (5/62, 8.06%) made no choices for the option "1". Right panel. Pre-instruction retest confidence ratings (indicated by black arrow) showed that 26 trainees (26/62, 41.94%) chose ITA as the best option, TTA as the next-best option with 2 points, and 15 trainees (15/62, 24.19%) with 3points; 1 trainee (1/62, 1.61%) could not make decision between ITA and TTA with 1 points; and 5 trainees (6/62, 8.06%) chose STA as the next-best option with 2 points, and 10 trainees (10/62, 16.13%) with 3 points; and 5 trainees (5/62, 8.06%) could not make decision between TTA and STA with 1 points. Consequently, 6 trainees (6/62, 9.68%) made no choices for the option "1". E. Left panel. Post-instruction initial confidence ratings (indicated by black arrow) showed that all trainees (62/62, 100%) chose ITA as the best option and TTA as the next-best option with 4 points. Right panel. Post-instruction retest confidence ratings (indicated by black arrow) showed that all trainees (62/62, 100%) chose ITA as the best option and TTA as the next-best option with 4 points. Magenta square frames with red arrows indicated all choices. BA = indicates basal artery; Cond. = condyle; For. = foramen; Hypogl. = hypoglossal; Inf. = inferior; ITA = inferior-tubercle approach; Jug. = jugular; Med., medulla; n = the number of subjects; Occi., occipital; PICA = posterior inferior cerebellar artery; Seg. = segment; STA = superior-tubercle approach; TTA = trans-tubercle approach; VA = vertebral artery; VI = abducens nerve; X = vagus nerve; XI = accessory nerve; XII = hypoglossal nerve; 3D = three-dimensional.


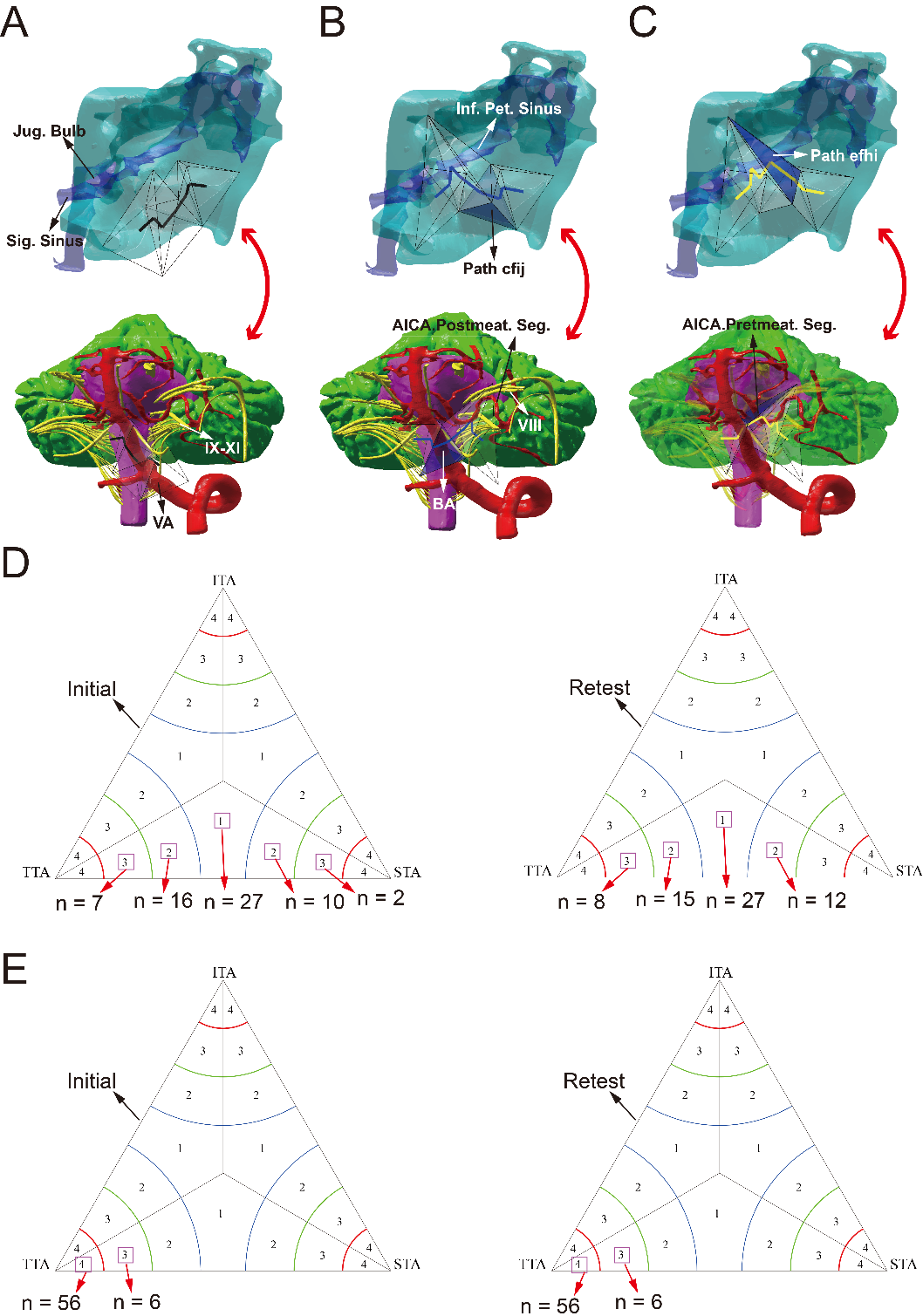


**Supplementary Figure 7.** A-C. The red two-direction arced arrow indicates two opposite sides of each surgical corridor in the TTA exemplar. A. A. The 3D rendering showed that the surgical corridor of ITA (The black lines in the transparent tetrahedrons) was remote from the non-dominant sigmoid sinus and the jugular bulb (indicated by black arrows). The grossly dilated VA (indicated by a black arrow) shifted towards the paths of ITA. The paths involved more volume of cranial nerves IX-XI (indicated by white arrows) for the lower location. B. The surgical corridor of TTA (The blue lines in the transparent tetrahedrons) approached the medial part of the inferior petrosal sinus (indicated by a white arrow). Next, it encountered the postmeatal segment of AICA (indicated by a black arrow). Then, it passed through the cleft between the cranial nerve VIII (indicated by a white arrow) and AICA. The path cfij of the corridor (transparent blue tetrahedron indicated by black arrow) formed a better operative space and avoided the VA; however, it involved the BA (indicated by white arrow). Finally, the corridor of TTA reached the inferior clivus with the same exposure as the corridor of ITA. C. The surgical corridor of STA (The yellow lines in the transparent tetrahedrons) showed that the path efhi (transparent blue tetrahedron indicated by white arrow) formed a narrow space and involved more premeatal segment of the AICA (indicated by black arrow). D. Left panel. Pre-instruction initial confidence ratings (indicated by black arrow) showed that 7 trainees (7/62, 11.29%) chose TTA as the best option, STA as the next-best option with 3 points, and 16 trainees (16/62, 25.81%) with 2 points; 2 trainees (2/62 3.23%) chose STA as the best option, TTA as the next-best option with 3 points, and 10 trainees (10/62, 16.13%) with 2 points; 27 trainees (27/62, 43.55%) made no choices for the option "1". Right panel. Pre-instruction retest confidence ratings (indicated by black arrow) showed that 8 trainees (8/62, 12.90%) chose TTA as the best option, STA as the next-best option with 3 points, and 15 trainees (15/62, 24.19%) with 2 points; 12 trainees (12/62 19.35%) chose STA as the best option, TTA as the next-best option with 2 points; 27 trainees (27/62, 43.55%) made no choices for the option "1". E. Left panel. Post-instruction initial confidence ratings (indicated by black arrow) showed that 56 trainees (56/62, 90.32%) chose TTA as the best option, STA as the next-best option with 4 points, and 6 trainees (6/62, 9.68%) with 3 points. Right panel. Post-instruction retest confidence ratings (indicated by black arrow) showed that 56 trainees (56/62, 90.32%) chose TTA as the best option, STA as the next-best option with 4 points, and 6 trainees (6/62, 9.68%) with 3 points. Magenta square frames with red arrows indicated all choices. AICA = indicates anterior inferior cerebellar artery; BA = basal artery; Inf. = inferior; ITA = inferior-tubercle approach; Jug. = jugular; n = the number of subjects; Pet. = petrosal; PICA = posterior inferior cerebellar artery; Postmeat. = postmeatal; Premeat., premeatal; Seg. = segment; Sig., sigmoid; STA = superior-tubercle approach; TTA = trans-tubercle approach; VA = vertebral artery; VIII = auditory nerve; IX = glossopharyngeal nerve; XI = accessory nerve; 3D = three-dimensional.

Supplementary Figure 8


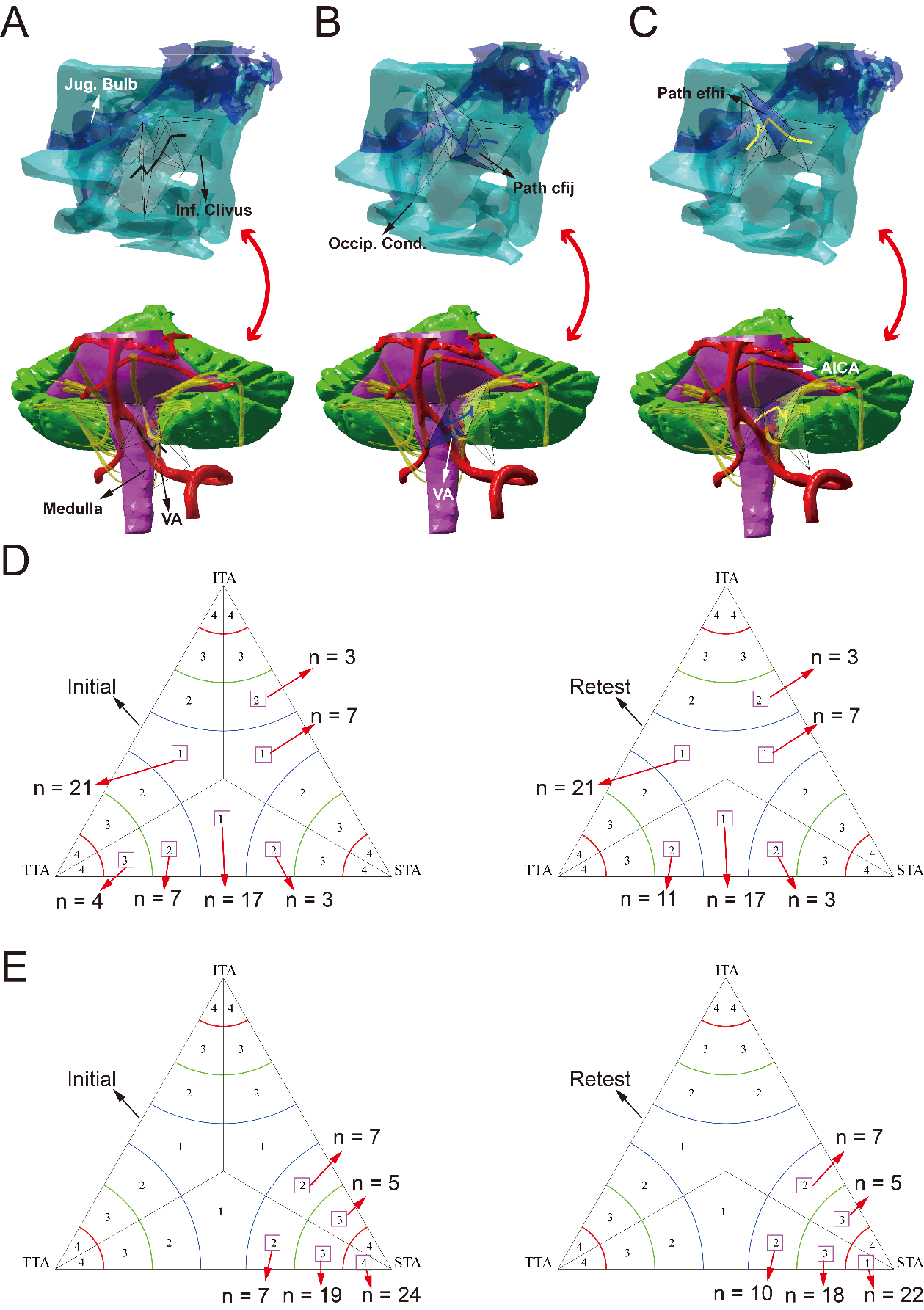


**Supplementary Figure 8.** A-C. The red two-direction arced arrow indicates two opposite sides of each surgical corridor in the STA exemplar. A. The 3D rendering showed that the surgical corridor of ITA (The black lines in the transparent tetrahedrons) exposed the inferior clivus (indicated by a black arrow). It was closer to the VA and medulla (black arrows) and more remote from the Jugular bulb (white arrow). B. The surgical corridor of TTA (The blue lines in the transparent tetrahedrons) started from the occipital condyle (indicated by a black arrow). It passed in an anterior and inferior direction with path cfij (transparent blue tetrahedron indicated by black arrow) involving the VA (indicated by white arrow). C. Compared to TTA, the path cfij was replaced by the path efhi (transparent blue tetrahedron indicated by black arrow) in STA. Path efhi avoided the VA and the variant AICA (indicated by white arrow). D. Left panel. Pre-instruction initial confidence ratings (indicated by black arrow) showed that 7 trainees (7/62, 11.29%) chose TTA as the best option, STA as the next-best option with 2 points, and 4 trainees (4/62, 6.45%) with 3 points; and 3 trainees (3/62, 4.84%) chose STA as the best option, TTA as the next-best option with 2 points; and 17 trainees (17/62, 27.42%) could not make decision between TTA and STA with 1 point; and 3 trainees (3/62, 4.84%) chose ITA as the best option, STA as the next-best option with 2 points; and 7 trainees (7/62, 11.29%) could not make decision between ITA and STA with 1 point; and 21 trainees (21/62, 33.87%) could not make decision between ITA and TTA with 1 point; Consequently, in total, 45 trainees (45/62, 72.58%) made no choices for the option "1" (count sum of the three areas [n=17+7+21]). Right panel. Pre-instruction retest confidence ratings (indicated by black arrow) showed that 11 trainees (11/62, 17.74%) chose TTA as the best option, STA as the next-best option with 2 points; and 3 trainees (3/62, 4.84%) chose STA as the best option, TTA as the next-best option with 2 points; and 17 trainees (17/62, 27.42%) could not make decision between TTA and STA with 1 point; and 3 trainees (3/62, 4.84%) chose ITA as the best option, STA as the next-best option with 2 points; and 7 trainees (7/62, 11.29%) could not make decision between ITA and STA with 1 point; and 21 trainees (21/62, 33.87%) could not make decision between ITA and TTA with 1 point; Consequently, in total, 45 trainees (45/62, 72.58%) made no choices for the option "1" (count sum of the three areas [n=17+7+21]). E. Left panel. Post-instruction initial confidence ratings (indicated by black arrow) showed that 24 trainees (24/62, 38.71%) chose STA as the best option, TTA as the next-best option with 4 points, and 19 trainees (19/62, 30.65%) with 3 points, and 7 trainees (7/62, 11.29%) with 2 points; and 5 trainees (5/62, 8.06%) chose STA as the best option, ITA as the next-best option with 3 points; and 7 trainees (7/62, 11.29%) with 2 points. Right panel. Post-instruction retest confidence ratings (indicated by black arrow) showed that 22 trainees (22/62, 35.48%) chose STA as the best option, TTA as the next-best option with 4 points, and 18 trainees (18/62, 29.03%) with 3 points, and 10 trainees (10/62, 16.13%) with 2 points; and 5 trainees (5/62, 8.06%) chose STA as the best option, ITA as the next-best option with 3 points; and 7 trainees (7/62, 11.29%) with 2 points. AICA indicates anterior inferior cerebellar artery; Cond. = condyle; Inf. = inferior; ITA = inferior-tubercle approach; Jug. = jugular; n = the number of subjects; Occi. = occipital; STA = superior-tubercle approach; TTA = trans-tubercle approach; VA = vertebral artery; 3D = three-dimensional.

Supplementary Figure 9


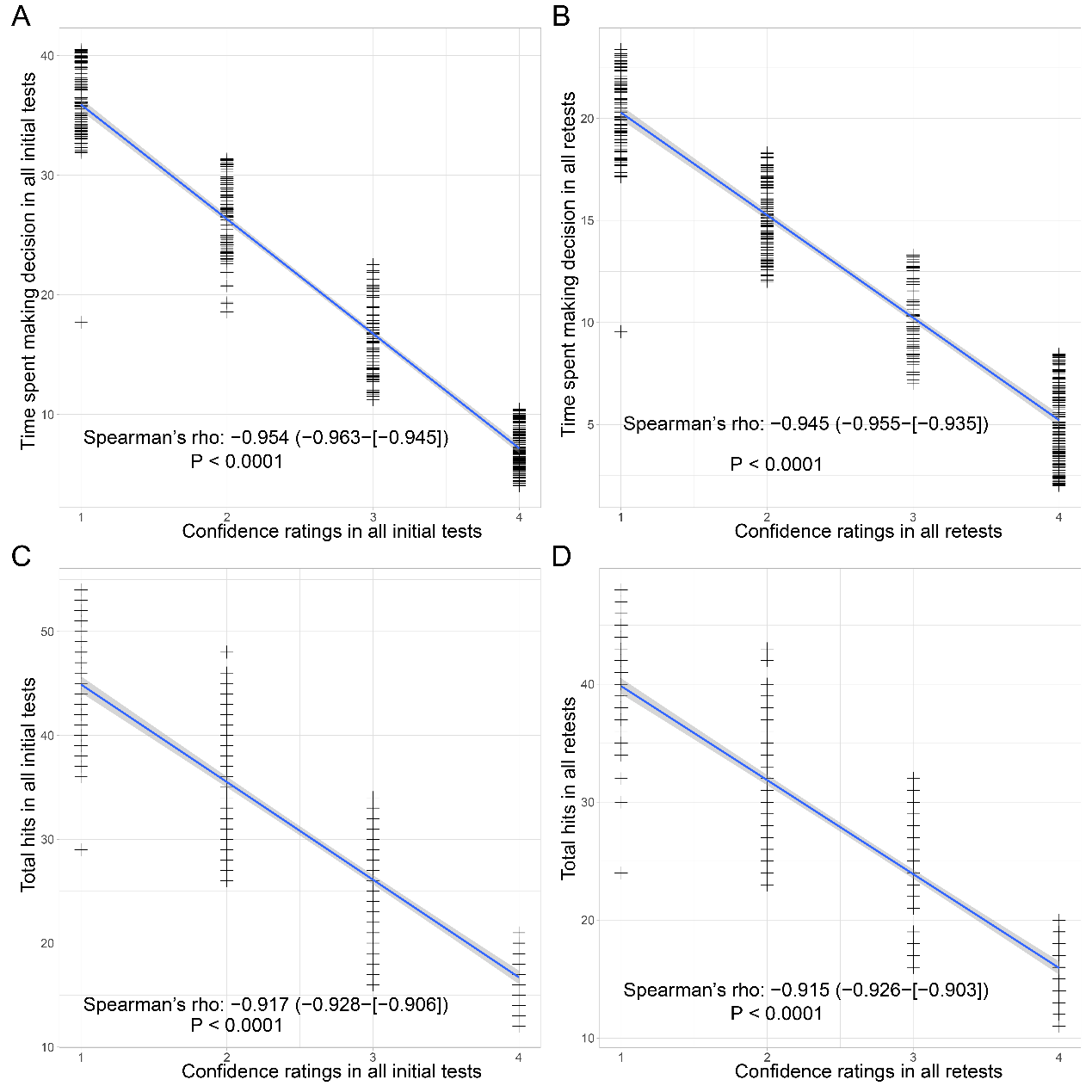


**Supplementary Figure 9.** Scatter plots of criterion validity correlation estimates between the confidence ratings and objective metrics (time spent making decision and total hits). The blue line denotes the best-fit linear correlation line. Grey shading shows 95% upper and lower confidence areas. A. Significant correlation between the confidence ratings and time spent making decision in all initial tests (sessions 1-3 and 7-9). B. Significant correlation between the confidence ratings and time spent making decision in all retests (sessions 4-6 and 10-12). C. Significant correlation between the confidence ratings and total hits in all initial tests (sessions 1-3 and 7-9). D. Significant correlation between the confidence ratings and total hits in all retests (sessions 4-6 and 10-12).

## Supplementary Tables

**Supplementary Table 1: Volumes of space and tissues measured in the twelve paths**

| **Items** | **Volumes measured for each path, median (IQR), n=50** | | | | | | | | | | | | |
| --- | --- | --- | --- | --- | --- | --- | --- | --- | --- | --- | --- | --- | --- |
|  | **abcd** | **abcg** | **bcdf** | **bcgh** | **bcfh** | **cdfj** | **befh** | **cfhi** | **cfij** | **efhi** | **efij** | **eijk** | **abcd** |
| Space, mm^3^ | 1096.89 (1047.53-1146.63) | 730.62 (722.18-740.96) | 395.84 (376.15-419.06) | 538.39 (509.4-559.18) | 349.06 (317.37-373.79) | 569.48 (546.23-593.54) | 244.85 (221.73-276.19) | 448.98 (409.54-502.88) | 457.97 (420-533.48) | 252.87 (225.61-272.56) | 144.54 (124.53-168.26) | 488.36 (447.89-518.82) | 1096.89 (1047.53-1146.63) |
| Bone, mm^3^ | 366.2 (324.05-385.02) | 376.36 (355.65-401.86) | 0.58 (0.42-0.82) | 81.61 (75.87-92.56) | 35.16 (24.93-43.69) | 33.81 (21.77-44.98) | 0 (0-0) | 150.91 (131.2-171.09) | 166.31 (133.53-181.55) | 0 (0-0) | 13.47 (10.51-17.5) | 70.47 (61.43-81.95) | 366.2 (324.05-385.02) |
| Cerebellum, mm^3^ | 30.29 (21.65-40.27) | 15.49 (8.94-21.47) | 0 (0-0) | 32.65 (23.83-42.49) | 3.54 (2.19-4.57) | 0 (0-0) | 0 (0-0) | 0 (0-0) | 0 (0-0) | 0 (0-0) | 0 (0-0) | 0 (0-0) | 30.29 (21.65-40.27) |
| Brainstem, mm^3^ | 1.53 (0.95-2.38) | 1.7 (0.96-2.73) | 35.75 (26.6-42.25) | 1.47 (0.79-2.31) | 2.14 (0.91-3.44) | 1.45 (0.79-2) | 26.28 (22.88-32.42) | 0 (0-0) | 0 (0-0) | 1.92 (0.92-2.74) | 2.38 (1.3-3.24) | 0 (0-0) | 1.53 (0.95-2.38) |
| Artery, mm^3^ | 33.49 (22.88-41.69) | 5.83 (4.07-8.17) | 6.43 (4.17-8.81) | 17.37 (10.93-23.12) | 7.29 (4.29-10.19) | 9.22 (5.68-13.6) | 5.02 (3.1-6.46) | 12.57 (9.24-16.17) | 21.57 (11.57-35.18) | 39.93 (31.87-45.36) | 3.36 (1.61-3.99) | 27.02 (21.55-34.59) | 33.49 (22.88-41.69) |
| Vein, mm^3^ | 0 (0-0) | 10.6 (7.78-15.82) | 0 (0-0) | 2.96 (1.56-3.98) | 0 (0-0) | 0 (0-0) | 0 (0-0) | 4.53 (2.65-6.31) | 0 (0-0) | 0.91 (0.41-1.56) | 0 (0-0) | 0 (0-0) | 0 (0-0) |
| Cranial nerve, mm^3^ | 70.57 (59.09-89.39) | 46.29 (26.32-55.36) | 5.01 (4.17-6.07) | 84.18 (73.88-121.74) | 14.54 (11.91-16.42) | 0.37 (0.15-0.55) | 8.01 (6.08-9.07) | 18.91 (14.18-22.11) | 1.38 (1.09-1.7) | 5.49 (3.23-7.86) | 1.1 (0.64-1.37) | 0 (0-0) | 70.57 (59.09-89.39) |

IQR = interquartile ranges; n = the number of subjects.

| **Paths** |  | **Weights calculated, median (IQR), n=50** | | | | | |
| --- | --- | --- | --- | --- | --- | --- | --- |
|  | **Plan 1** | **Plan 2** | **Plan 3** | **Plan 4** | **Plan 5** | **Plan 6** | **Plan 7** |
| abcd | 709.66 (570.91-834.37) | 709.66 (570.91-834.37) | 709.66 (570.91-834.37) | 709.66 (570.91-834.37) | NA | NA | NA |
| abcg | NA | NA | NA | NA | 413.8 (309.37-468.08) | 413.8 (309.37-468.08) | 413.8 (309.37-468.08) |
| bcdf | 443.59 (379.77-511.52) | 443.59 (379.77-511.52) | 443.59 (379.77-511.52) | 443.59 (379.77-511.52) | NA | NA | NA |
| bcgh | NA | NA | NA | NA | 677.72 (590.32-806.76) | 677.72 (590.32-806.76) | 677.72 (590.32-806.76) |
| bcfh | NA | 163.06 (132.47-191.42) | 163.06 (132.47-191.42) | 163.06 (132.47-191.42) | 163.06 (132.47-191.42) | 163.06 (132.47-191.42) | 163.06 (132.47-191.42) |
| cdfj | 102.43 (63.36-147.5) | NA | NA | NA | NA | NA | NA |
| befh | NA | NA | NA | 360.5 (294.48-419.85) | NA | NA | 360.5 (294.48-419.85) |
| cfhi | NA | 257.94 (226.89-290.72) | 257.94 (226.89-290.72) | NA | 257.94 (226.89-290.72) | 257.94 (226.89-290.72) | NA |
| cfij | 218.87 (119.61-353.62) | 218.87 (119.61-353.62) | NA | NA | 218.87 (119.61-353.62) | NA | NA |
| efhi | NA | NA | 453.8 (370.09-517.38) | 453.8 (370.09-517.38) | NA | 453.8 (370.09-517.38) | 453.8 (370.09-517.38) |
| efij | 58.42 (41.54-71.26) | 58.42 (41.54-71.26) | 58.42 (41.54-71.26) | 58.42 (41.54-71.26) | 58.42 (41.54-71.26) | 58.42 (41.54-71.26) | 58.42 (41.54-71.26) |
| eijk | 264.88 (210.54-340.72) | 264.88 (210.54-340.72) | 264.88 (210.54-340.72) | 264.88 (210.54-340.72) | 264.88 (210.54-340.72) | 264.88 (210.54-340.72) | 264.88 (210.54-340.72) |
| The sum for the plan | 1787.24 (1623.35-2022.84) | 2101.47 (1965.61-2296.75) | 2348.65 (2112.46-2508.19) | 2446.69 (2229.86-2592.03) | 2081.48 (1893.79-2285.63) | 2301.39 (2045.86-2481.07) | 2380.5 (2220.9-2633.57) |

**Supplementary Table 2: Weights calculated for the twelve paths of the seven plans**

IQR = interquartile ranges; n = the number of subjects; NA = not applicable.

**Supplementary Table 3****: Parameters and comparative results for the seven plans**

| **Items** | **Parameters in each plan, median (IQR), n=50** | | | | | | | **Kruskal-Wallis test *P* value** |
| --- | --- | --- | --- | --- | --- | --- | --- | --- |
|  | **Plan 1** | **Plan 2** | **Plan 3** | **Plan 4** | **Plan 5** | **Plan 6** | **Plan 7** |  |
| Space **^*^**, mm^3^ | 3162.46 (3060.74-3257.95) | 3389.3 (3279.6-3503.42) | 3179.11 (3104.81-3243.84) | 2963.25 (2903.37-3018.44) | 3167.26 (3065.28-3268.63) | 2956.26 (2886.24-2995.82) | 2740.48 (2684.41-2796.89) | < 0.0001 |
| Bone ^†,^ mm^3^ | 635.12 (601.63-679.58) | 791.26 (745.32-832.58) | 637.68 (586.40-674.71) | 488.37 (432.42-520.62) | 883.54 (850.07-941.68) | 733.43 (701.14-771.08) | 575.53 (554.50-606.44) | < 0.0001 |
| Cerebellum ^‡^, mm^3^ | 30.29 (21.65-40.27) | 33.61 (26.13-43.50) | 33.61 (26.13-43.50) | 33.61 (26.13-43.50) | 50.70 (38.49-63.72) | 50.70 (38.49-63.72) | 50.70 (38.49-63.72) | < 0.0001 |
| Brainstem ^§^, mm^3^ | 41 (33.36-48.27) | 41.66 (33.84-47.82) | 43.01 (37.16-49.95) | 70.95 (64.60-77.32) | 7.82 (5.89-9.86) | 9.89 (8.04-11.78) | 36.66 (32.69-41.13) | < 0.0001 |
| Artery ^¶^, mm^3^ | 100.85 (85.2-120.14) | 115.24 (98.03-130.86) | 131.04 (114.58-141.26) | 121.09 (107.13-134.44) | 98.49 (78.97-112.98) | 111.38 (102.13-123.86) | 104.80 (93.36-116.95) | < 0.001 |
| Vein, mm^3^ | 0 (0-0) | 4.53 (2.65-6.31) | 5.72 (3.95-7.30) | 0.91(0.40-1.56) | 18.23 (14.07-24.53) | 19.54 (15.66-25.45) | 15.32 (10.47-20.34) | < 0.0001 |
| CNs, mm^3^ | 79.65 (65.94-97.4) | 110.98 (99.2-127.17) | 114.37 (104.10-129.88) | 103.66 (91.36-121.82) | 168.14 (150.82-192.74) | 173.36 (151.69-195.06) | 166.47 (137.50-187.91) | < 0.0001 |
| Weight ^£^ | 1787.24 (1623.35-2022.84) | 2101.47 (1965.61-2296.75) | 2348.65 (2112.46-2508.19) | 2446.69 (2229.86-2592.03) | 2081.48 (1893.79-2285.63) | 2301.39 (2045.86-2481.07) | 2380.50 (2220.90-2633.57) | < 0.0001 |

IQR = interquartile ranges; n = the number of subjects.

Unless otherwise indicated, the Wilcoxon matched-pairs test showed a statistically significant difference for each pairwise comparison.

**^*^** The Wilcoxon matched-pairs test failed to show statistically significant variation between plan 1 and plan 3 (3162.46 [3060.74-3257.95] vs. 3179.11 [3104.81-3243.84], *P* > .999), plan 1 and plan 5 (3162.46 [3060.74-3257.95] vs. 3167.26 [3065.28-3268.63], *P* > .999), plan 3 and plan 5 (3179.11 [3104.81-3243.84] vs. 3167.26 [3065.28-3268.63], *P* > .999), and plan 4 and plan 6 (2963.25 [2903.37-3018.44] vs. 2956.26 [2886.24-2995.82], *P* > .999).

^†^ The Wilcoxon matched-pairs test failed to show statistically significant variation between plan 1 and plan 3 (635.12 [601.63-679.58] vs. 637.68 [586.40-674.71], *P* > .999).

^‡^ The cerebellum volume in plan 2, plan 3, and plan 4 was the same, and in plan 5, plan 6, and plan 7 were the same.

^§^ The Wilcoxon matched-pairs test failed to show statistically significant variation between plan 1 and plan 2 (41 [33.36-48.27] vs. 41.66 [33.84-47.82], *P* = .227), plan 1 and plan 7 (41 [33.36-48.27] vs. 36.66 [32.69-41.13], *P* > .999), and plan 2 and plan 7 (41.66 [33.84-47.82] vs. 36.66 [32.69-41.13], *P* = .554).

^¶^ The Wilcoxon matched-pairs test failed to show statistically significant variation between plan 1 and plan 5 (100.85 [85.2-120.14] vs. 98.49 [78.97-112.98], *P* = .528), plan 1 and plan 6 (100.85 [85.2-120.14] vs. 111.38 [102.13-123.86], *P* = .146), plan 1 and plan 7 (100.85 [85.2-120.14] vs. 104.80 [93.36-116.95], *P* > .999), plan 2 and plan 4 (115.24 [98.03-130.86] vs. 121.09 [107.13-134.44], *P* = .053), plan 2 and plan 7 (115.24 [98.03-130.86] vs. 104.80 [93.36-116.95], *P* = .104), and plan 5 and plan 7 (98.49 [78.97-112.98] vs. 104.80 [93.36-116.95], *P* = .053)

^£^ The Wilcoxon matched-pairs test failed to show statistically significant variation between plan 2 and plan 5 (2101.47 [1965.61-2296.75] vs. 2081.48 [1893.79-2285.63], *P* > .999), plan 2 and plan 6 (2101.47 [1965.61-2296.75] vs. 2301.39 [2045.86-2481.07], *P* = .051), plan 3 and plan 6 (2348.65 [2112.46-2508.19] vs. 2301.39 [2045.86-2481.07], *P* > .999), plan 3 and plan 7 (2348.65 [2112.46-2508.19] vs. 2380.50 [2220.90-2633.57], *P* > .999), and plan 4 and plan 7 (2446.69 [2229.86-2592.03] vs. 2380.50 [2220.90-2633.57], *P* > .999).

**Supplementary Table 4. Dijkstra algorithm for path planning**

| **Steps of the Dijkstra algorithm for path planning** |
| --- |
| Require: A priority set of nodes U and a current set of nodes S |
| N = the number of nodes |
| Initial stage: S = {start node}, U = {remaining N nodes} |
| The current sum of weights of paths = minimum sum of weights of paths between the start node and a new node in S |
| The new weight of path = the weight of path between the above new node in S and a neighboring node in U |
| Priority sum of weights of paths = minimum sum of weights of paths between the start node and the neighboring node |
| While N-1 > 0 |
| Identify all weights of current and priority paths |
| If the current sum of weights of paths + new weight of path < priority sum of weights of paths |
| Priority sum of weights of paths = current sum of weights of paths + new weight of a path |
| End if |
| For a neighboring node in U, do |
| If the priority sum of weights of paths is found |
| Remove the neighboring node from U |
| New node = the neighboring node |
| S = S ∪ new node |
| The current sum of weights of paths = priority sum of weights of paths |
| End if |
| End for |
| N = N-1 |
| End while |
| Find the optimal queue of path planning according to the current sum of weights of paths |

**Supplementary Table 5. Evaluation of decision capacity and confidence for each training session**

| **Sessions** | **Evaluation of decision capacity and confidence, median (IQR), n=62** | | |
| --- | --- | --- | --- |
|  | **Time spent making decision^*^, mins.** | **Total hits**^†^ | **Confidence ratings**^‡^ |
| Session 1 (Pre-instruction initial test of ITA exemplar) | 24.02 (18.96, 28.14) | 30 (25, 39) | 2 (2, 3) |
| Session 2 (Pre-instruction initial test of TTA exemplar) | 30.24 (26.45, 35.10) | 37 (31, 45) | 2 (1, 2) |
| Session 3 (Pre-instruction initial test of STA exemplar) | 34.21 (31.01, 37.41) | 42 (38, 46) | 1 (1, 2) |
| Session 4 (Pre-instruction retest of ITA exemplar) | 14.80 (11.90, 16.72) | 28 (24, 32) | 2 (2, 3) |
| Session 5 (Pre-instruction retest of TTA exemplar) | 17.41 (14.41, 19.31) | 34 (28, 39) | 2 (1, 2) |
| Session 6 (Pre-instruction retest of STA exemplar) | 19.36 (17.69, 21.56) | 39 (34, 42) | 1 (1, 2) |
| Session 7 (Post-instruction initial test of ITA exemplar) | 7.13 (5.58, 8.76) | 16 (15, 18) | 4 (4, 4) |
| Session 8 (Post-instruction initial test of TTA exemplar) | 7.06 (5.94, 9.33) | 17 (16, 19) | 4 (4, 4) |
| Session 9 (Post-instruction initial test of STA exemplar) | 12.82 (8.36, 16.84) | 28 (17, 33) | 3 (3, 4) |
| Session 10 (Post-instruction retest of ITA exemplar) | 5.22 (3.14, 6.87) | 16 (14, 17) | 4 (4, 4) |
| Session 11 (Post-instruction retest of TTA exemplar) | 5.80 (3.51, 7.57) | 16 (14, 18) | 4 (4, 4) |
| Session 12 (Post-instruction retest of STA exemplar) | 9.86 (6.67, 13.07) | 27 (17, 37) | 3 (2, 4) |
| Kruskal-Wallis test *P* value | <0.0001 | <0.0001 | <0.0001 |

IQR = interquartile ranges; ITA = inferior-tubercle approach; mins = minutes; n = the number of subjects; STA = superior-tubercle approach; TTA = trans-tubercle approach.

Unless otherwise indicated, the Wilcoxon matched-pairs test showed a statistically significant difference for each pairwise comparison.

**^*^** The Wilcoxon matched-pairs test failed to show statistically significant variation between session 2 and session 3 (30.24 [26.45-35.10] vs. 34.21 [31.01-37.41], *P* = 0.186), session 7 and session 8 (7.13 [5.58-8.76] vs. 7.06 [5.94-9.33], *P* > 0.999), session 10 and session 11 (5.22 [3.14-6.87] vs. 5.80 [3.51-7.57], *P* > 0.999).

^†^ The Wilcoxon matched-pairs test failed to show statistically significant variation between session 1 and session 4 (30 [25-39] vs. 28 [24-32], *P* = 0.362), session 2 and session 3 (37 [31-45] vs. 42 [38-46], *P* > 0.999), session 3 and session 6 (42 [38-46] vs. 39 [34-42], *P* = 0.058), session 7 and session 8 (16 [15-18] vs. 17 [16-19], *P* > 0.999), session 7 and session 10 (16 [15-18] vs. 16 [14-17], *P* > 0.999), session 8 and session 11 (17 [16-19] vs. 16 [14-18], *P* > 0.999), session 9 and session 12 (28 [17-33] vs. 27 [17-37], *P* > 0.999), session 10 and session 11 (16 [14-17] vs. 16 [14-18], *P* > 0.999).

^‡^ The Wilcoxon matched-pairs test failed to show statistically significant variation between session 1 and session 4 (2 [2-3] vs. 2 [2-3], *P* = 0.503), session 2 and session 5 (2 [1-2] vs. 2 [1-2], *P* > 0.999), session 3 and session 6 (1 [1-2] vs. 1 [1-2], *P* > 0.999), session 7 and session 8 (4 [4-4] vs. 4 [4-4], *P* > 0.999), session 7 and session 10 (4 [4-4] vs. 4 [4-4], *P* > 0.999), session 8 and session 11 (4 [4-4] vs. 4 [4-4], *P* > 0.999), session 9 and session 12 (3 [3-4] vs. 3 [2-4], *P* > 0.999), session 10 and session 11 (4 [4-4] vs. 4 [4-4], *P* > 0.999).

**Supplementary Table 6. Association between trainee’s characteristics and time spent making decision**

| **Sessions** | **Time spent making decision, mins, median (IQR), n=62** | | | | **Kruskal-Wallis test *P* value** | **Time spent making decision, mins, median (IQR), n=62** | | **Unpaired Mann-Whitney U test *P* value** | **Time spent making decision, mins, median (IQR), n=62** | | **Unpaired Mann-Whitney U test *P* value** |
| --- | --- | --- | --- | --- | --- | --- | --- | --- | --- | --- | --- |
|  | **26 yrs** | **27 yrs** | **28 yrs** | **29 yrs** |  | **Male** | **Female** |  | **Neurosurgery** | **Otolaryngology** |  |
| Session 1**^*^** | 28.11 (25.35, 33.40) | 20.80 (18.31, 27.15) | 19.79 (16.34, 23.77) | 27.07 (23.40, 29.46) | 0.004 | 22.98 (17.55, 27.12) | 24.48 (20.80, 28.34) | 0.264 | 24.97 (20.31, 28.36) | 23.44 (17.53, 28.12) | 0.329 |
| Session 2 | 26.85 (23.73, 30.81) | 32.06 (26.73, 36.08) | 29.69 (24.81, 34.94) | 31.00 (27.93, 34.26) | 0.475 | 29.07 (25.17, 34.77) | 31.63 (26.93, 35.38) | 0.412 | 31.89 (26.64, 35.74) | 29.37 (24.65, 33.68) | 0.315 |
| Session 3 | 32.79 (27.96, 38.22) | 34.48 (32.40, 37.44) | 35.65 (32.67, 37.82) | 32.92 (30.64, 34.26) | 0.424 | 34.45 (30.37, 37.00) | 34.02 (31.22, 37.71) | 0.966 | 34.42 (31.33, 37.21) | 34.20 (30.90, 37.44) | 0.830 |
| Session 4 | 14.99 (14.25, 19.48) | 14.77 (10.84, 16.73) | 13.30 (10.31, 15.67) | 16.36 (12.76, 16.92) | 0.270 | 14.70 (10.32, 16.64) | 15.66 (12.64, 16.76) | 0.298 | 15.67 (13.85, 16.73) | 14.32 (10.35, 16.03) | 0.189 |
| Session 5^†^ | 15.96 (13.28, 18.97) | 17.79 (14.84, 19.05) | 17.46 (15.57, 19.91) | 16.05 (13.34, 18.53) | 0.724 | 17.13 (14.38, 19.46) | 17.87 (14.75, 19.05) | 0.734 | 18.37 (15.31, 21.41) | 16.60 (13.21, 17.98) | 0.015 |
| Session 6 | 18.44 (16.45, 21.13) | 19.71 (18.17, 21.36) | 20.87 (18.05, 21.66) | 19.05 (14.25, 20.48) | 0.548 | 19.67 (17.81, 21.66) | 19.07 (16.88, 21.35) | 0.347 | 18.93 (17.70, 21.64) | 19.43 (17.18, 21.44) | 0.931 |
| Session 7^‡^ | 6.84 (5.20, 8.49) | 7.16 (5.85, 8.73) | 7.10 (6.20, 9.40) | 7.05 (5.90, 8.74) | 0.817 | 7.88 (6.05, 9.44) | 6.36 (5.11, 8.33) | 0.054 | 7.97 (6.29, 9.45) | 6.71 (5.17, 8.45) | 0.036 |
| Session 8 | 6.98 (6.27, 8.73) | 6.77 (5.40, 9.09) | 7.88 (5.97, 9.88) | 7.65 (6.26, 8.77) | 0.693 | 7.76 (6.14, 9.33) | 6.69 (5.66, 9.25) | 0.373 | 7.27 (5.97, 9.60) | 7.03 (5.93, 9.27) | 0.909 |
| Session 9 | 8.84 (7.05, 15.48) | 11.69 (9.15, 15.58) | 15.26 (9.94, 22.93) | 12.69 (8.35, 14.11) | 0.147 | 13.07 (8.80, 18.67) | 11.79 (7.94, 16.55) | 0.381 | 13.18 (9.62, 16.76) | 11.81 (7.78, 16.86) | 0.326 |
| Session 10 | 4.61 (3.88, 6.59) | 6.21 (3.43, 7.75) | 4.54 (2.19, 6.54) | 5.09 (2.91, 6.52) | 0.378 | 4.57 (2.98, 6.54) | 6.15 (4.21, 7.61) | 0.123 | 5.49 (3.44, 7.08) | 4.82 (2.92, 6.65) | 0.413 |
| Session 11 | 4.42 (3.40, 5.65) | 6.18 (3.87, 7.57) | 5.58 (2.46, 7.26) | 6.34 (5.49, 8.43) | 0.360 | 5.68 (3.51, 7.14) | 6.00 (3.59, 7.61) | 0.054 | 6.34 (3.64, 7.64) | 5.12 (3.33, 7.50) | 0.430 |
| Session 12 | 8.18 (6.38, 11.90) | 10.86 (6.64, 13.06) | 12.15 (7.33, 14.74) | 8.73 (6.49, 12.86) | 0.435 | 11.97 (6.74, 14.38) | 8.48 (6.80, 12.96) | 0.246 | 11.02 (6.95, 13.29) | 9.64 (6.33, 13.07) | 0.741 |

IQR = interquartile ranges; mins = minutes; n = the number of subjects.

The unpaired Mann-Whitney U test failed to show statistical significance for each comparison unless otherwise indicated.

**^*^** The unpaired Mann-Whitney U test showed statistically significant variation between 26yrs and 28yrs (28.11 [25.35-33.40] vs. 19.79 [16.34-23.77], *P* = 0.013).

^†^ The unpaired Mann-Whitney U test showed statistically significant variation between neurosurgery and otolaryngology (18.37 [15.31-21.41] vs. 16.60 [13.21, 17.98], *P* = 0.015).

^‡^ The unpaired Mann-Whitney U test showed statistically significant variation between neurosurgery and otolaryngology (7.97 [6.29-9.45] vs. 6.71 [5.17-8.45], *P* = 0.036).

**Supplementary Table 7. Association between trainee’s characteristics and total hits**

| **Sessions** | **Total hits, median (IQR), n=62** | | | | **Kruskal-Wallis test *P* value** | **Total hits, median (IQR), n=62** | | **Unpaired Mann-Whitney U test *P* value** | **Total hits, median (IQR), n=62** | | **Unpaired Mann-Whitney U test *P* value** |
| --- | --- | --- | --- | --- | --- | --- | --- | --- | --- | --- | --- |
|  | **26 yrs** | **27 yrs** | **28 yrs** | **29 yrs** |  | **Male** | **Female** |  | **Neurosurgery** | **Otolaryngology** |  |
| Session 1 | 38 (28, 43) | 29 (24, 34) | 29 (21, 36) | 33 (31, 38) | 0.168 | 29 (24, 38) | 32 (28, 39) | 0.282 | 29 (25, 40) | 30 (26, 38) | 0.677 |
| Session 2 | 31 (27, 41) | 37 (32, 45) | 38 (32, 42) | 38 (35, 46) | 0.502 | 36 (30, 44) | 40 (35, 45) | 0.137 | 37 (31, 46) | 37 (32, 42) | 0.595 |
| Session 3 | 39 (31, 41) | 42 (39, 47) | 44 (40, 47) | 43 (36, 47) | 0.123 | 42 (38, 46) | 43 (36, 46) | 0.871 | 43 (38, 47) | 40 (38, 46) | 0.168 |
| Session 4**^*^** | 34 (29, 37) | 25 (23, 31) | 27 (22, 31) | 30 (28, 32) | 0.010 | 28 (24, 32) | 30 (26, 32) | 0.472 | 30 (26, 35) | 27 (24, 31) | 0.071 |
| Session 5 | 33 (29, 37) | 36 (30, 39) | 35 (29, 41) | 30 (27, 39) | 0.792 | 33 (29, 38) | 36 (27, 39) | 0.843 | 35 (31, 41) | 32 (26, 38) | 0.074 |
| Session 6 | 36 (33, 42) | 39 (36, 42) | 37 (35, 43) | 38 (33, 41) | 0.694 | 40 (35, 42) | 37 (34, 41) | 0.185 | 38 (35, 42) | 39 (34, 41) | 0.429 |
| Session 7 | 17 (16, 18) | 15 (14, 18) | 16 (16, 17) | 17 (16, 18) | 0.241 | 17 (16, 18) | 16 (15, 18) | 0.210 | 16 (15, 18) | 16 (15, 17) | 0.636 |
| Session 8 | 18 (16, 19) | 17 (15, 18) | 18 (16, 19) | 17 (16, 19) | 0.300 | 17 (16, 19) | 17 (16, 18) | 0.339 | 17 (16, 19) | 17 (16, 19) | 0.772 |
| Session 9 | 19 (17, 27) | 27 (17, 33) | 31 (19, 44) | 30 (19, 31) | 0.141 | 28 (19, 38) | 28 (16, 32) | 0.201 | 31 (19, 33) | 27 (16, 32) | 0.188 |
| Session 10 | 16 (15, 16) | 15 (14, 17) | 15 (14, 17) | 17 (16, 18) | 0.350 | 15 (14, 17) | 16 (15, 17) | 0.471 | 15 (14, 17) | 16 (14, 17) | 0.473 |
| Session 11 | 16 (14, 18) | 16 (14, 17) | 17 (15, 18) | 17 (16, 19) | 0.320 | 16 (14, 18) | 16 (15, 18) | 0.507 | 17 (14, 18) | 16 (14, 17) | 0.577 |
| Session 12 | 18 (15, 28) | 27 (15, 34) | 31 (19, 40) | 29 (25, 31) | 0.199 | 27 (18, 37) | 27 (16, 31) | 0.388 | 28 (18, 37) | 27 (16, 36) | 0.585 |

IQR = interquartile ranges; mins = minutes; n = the number of subjects.

The unpaired Mann-Whitney U test failed to show statistical significance for each comparison unless otherwise indicated.

**^*^** The unpaired Mann-Whitney U test showed statistically significant variation between 26yrs and 27yrs (34 [29-37] vs. 25 [23-31], *P* = 0.024).

**Supplementary Table 8. Association between trainee’s characteristics and confidence ratings**

| **Sessions** | **Confidence ratings, median (IQR), n=62** | | | | **Kruskal-Wallis test *P* value** | **Confidence ratings, median (IQR), n=62** | | **Unpaired Mann-Whitney U test *P* value** | **Confidence ratings, median (IQR), n=62** | | **Unpaired Mann-Whitney U test *P* value** |
| --- | --- | --- | --- | --- | --- | --- | --- | --- | --- | --- | --- |
|  | **26 yrs** | **27 yrs** | **28 yrs** | **29 yrs** |  | **Male** | **Female** |  | **Neurosurgery** | **Otolaryngology** |  |
| Session 1 | 2 (1, 2) | 2 (2, 3) | 3 (2, 3) | 2 (2, 2) | 0.024 | 3 (2, 3) | 2 (2, 3) | 0.088 | 2 (2, 3) | 2 (2, 3) | 0.624 |
| Session 2 | 2 (1, 2) | 1 (1, 2) | 2 (1, 2) | 2 (1, 2) | 0.700 | 2 (1, 2) | 2 (1, 2) | 0.305 | 1 (1, 2) | 2 (1, 2) | 0.110 |
| Session 3 | 2 (1, 2) | 1 (1, 1) | 1 (1, 1) | 1 (1, 2) | 0.151 | 1 (1, 2) | 1 (1, 2) | 0.842 | 1 (1, 2) | 1 (1, 2) | 0.985 |
| Session 4**^*^** | 2 (1, 2) | 2 (2, 3) | 2 (2, 3) | 2 (2, 2) | 0.025 | 2 (2, 3) | 2 (2, 2) | 0.177 | 2 (2, 2) | 2 (2, 3) | 0.475 |
| Session 5 | 2 (1, 2) | 1 (1, 2) | 2 (1, 2) | 2 (1, 2) | 0.450 | 2 (1, 2) | 2 (1, 2) | 0.704 | 1 (1, 2) | 1 (1, 2) | 0.074 |
| Session 6 | 2 (1, 2) | 1 (1, 1) | 1 (1, 1) | 1 (1, 2) | 0.129 | 1 (1, 2) | 1 (1, 2) | 0.855 | 1 (1, 2) | 1 (1, 2) | 0.933 |
| Session 7^†^ | 4 (4, 4) | 4 (4, 4) | 4 (4, 4) | 4 (4, 4) | NA | 4 (4, 4) | 4 (4, 4) | NA | 4 (4, 4) | 4 (4, 4) | NA |
| Session 8 | 4 (4, 4) | 4 (4, 4) | 4 (4, 4) | 4 (4, 4) | 0.577 | 4 (4, 4) | 4 (4, 4) | 0.543 | 4 (4, 4) | 4 (4, 4) | 0.218 |
| Session 9 | 4 (3, 4) | 3 (3, 4) | 3 (2, 4) | 3 (3, 4) | 0.110 | 3 (2, 4) | 3 (3, 4) | 0.244 | 3 (3, 4) | 3 (3, 4) | 0.470 |
| Session 10^‡^ | 4 (4, 4) | 4 (4, 4) | 4 (4, 4) | 4 (4, 4) | NA | 4 (4, 4) | 4 (4, 4) | NA | 4 (4, 4) | 4 (4, 4) | NA |
| Session 11 | 4 (4, 4) | 4 (4, 4) | 4 (4, 4) | 4 (4, 4) | 0.687 | 4 (4, 4) | 4 (4, 4) | 0.804 | 4 (4, 4) | 4 (4, 4) | 0.716 |
| Session 12 | 4 (3, 4) | 3 (3, 4) | 3 (2, 4) | 3 (3, 3) | 0.215 | 3 (2, 4) | 3 (3, 4) | 0.229 | 3 (2, 4) | 3 (2, 4) | 0.731 |

IQR = interquartile ranges; mins = minutes; n = the number of subjects; NA = not applicable.

The unpaired Mann-Whitney U test failed to show statistical significance for each comparison unless otherwise indicated.

**^*^** The unpaired Mann-Whitney U test showed statistically significant variation between 26yrs and 28yrs (2 [1-2] vs. 2 [2-3], *P* = 0.042).

^†^ The confidence ratings were the same for all trainees.

^‡^ The confidence ratings were the same for all trainees.

**Supplementary Table 9. Hit percentages of concerned items in each training session**

| **Sessions** | **Hit percentages of concerned items, %, median (IQR), n=62** | | | | | | | **Kruskal-Wallis test *P* value** |
| --- | --- | --- | --- | --- | --- | --- | --- | --- |
|  | **Operative space** | **Bone** | **Cerebellum** | **Brainstem** | **Artery** | **Vein** | **CNs** |  |
| Session 1 | 19.61 (16.67, 22.69) | 12.50 (10.28, 14.21) | 10.53 (8.70, 13.63) | 10.62 (8.38, 12.74) | 13.92 (12.50, 15.38) | 16.00 (14.29, 17.81) | 15.79 (13.79, 18.61) | <0.0001 |
| Session 2 | 26.43 (23.16, 29.91) | 11.24 (9.45, 13.29) | 9.68 (7.96, 12.74) | 9.52 (7.48, 11.87) | 13.42 (10.88, 15.00) | 14.81 (12.94, 16.67) | 14.09 (11.31, 17.20) | <0.0001 |
| Session 3 | 25.51 (23.12, 29.25) | 11.70 (9.62, 13.29) | 10.53 (7.69, 12.44) | 10.17 (7.48, 11.87) | 12.90 (11.63, 14.29) | 14.67 (13.07, 16.28) | 14.71 (12.78, 17.21) | <0.0001 |
| Session 4 | 15.51 (13.68, 16.09) | 12.90 (11.11, 15.24) | 11.11 (8.70, 13.33) | 12.50 (11.11, 13.83) | 15.07 (12.50, 17.02) | 15.63 (12.21, 17.77) | 17.14 (14.73, 20.00) | <0.0001 |
| Session 5 | 23.61 (21.09, 26.62) | 11.65 (9.52, 13.46) | 10.34 (7.69, 12.50) | 11.43 (9.56, 12.88) | 12.86 (11.11, 15.95) | 13.84 (11.46, 16.67) | 15.71 (12.38, 18.88) | <0.0001 |
| Session 6 | 22.61 (20.68, 25.00) | 11.90 (10.33, 13.95) | 10.13 (7.69, 12.40) | 11.48 (10.06, 12.50) | 13.73 (11.43, 15.38) | 14.12 (11.38, 17.02) | 15.60 (13.01, 18.43) | <0.0001 |
| Session 7 | 22.22 (21.05, 25.00) | 9.43 (6.25, 13.33) | 7.69 (6.25, 12.50) | 12.91 (11.76, 15.69) | 16.23 (11.27, 20.00) | 12.50 (10.53, 15.79) | 16.67 (12.50, 20.00) | <0.0001 |
| Session 8 | 35.29 (32.33, 37.50) | 6.91 (5.88, 10.97) | 7.14 (5.88, 11.11) | 11.44 (7.69, 12.50) | 12.91 (7.69, 15.79) | 11.33 (6.79, 13.22) | 13.81 (8.27, 16.67) | <0.0001 |
| Session 9 | 31.25 (28.30, 36.84) | 7.02 (6.25, 11.87) | 7.02 (6.25, 11.11) | 11.76 (10.62, 13.22) | 12.50 (8.19, 17.59) | 11.23 (9.68, 13.22) | 12.70 (11.43, 17.21) | <0.0001 |
| Session 10 | 23.08 (21.43, 25.00) | 13.33 (11.11, 15.79) | 7.14 (6.25, 11.11) | 10.53 (6.79, 13.12) | 14.29 (11.76, 17.65) | 13.33 (11.76, 15.79) | 16.67 (14.29, 19.69) | <0.0001 |
| Session 11 | 37.50 (35.29, 39.78) | 11.11 (7.21, 12.50) | 6.67 (5.88, 7.69) | 7.14 (6.25, 11.11) | 11.76 (7.85, 14.29) | 11.44 (7.21, 12.91) | 13.19 (11.76, 15.38) | <0.0001 |
| Session 12 | 33.33 (29.63, 35.71) | 11.65 (7.85, 14.29) | 7.14 (5.97, 9.22) | 7.85 (7.14, 11.43) | 12.66 (10.00, 14.81) | 11.76 (7.85, 13.33) | 14.90 (12.50, 17.43) | <0.0001 |

CN = cranial nerve; IQR = interquartile ranges; n = the number of subjects.

Unless otherwise indicated, the Wilcoxon matched-pairs test showed a statistically significant difference for each pairwise comparison.

For session 1, the Wilcoxon matched-pairs test failed to show statistically significant variation between bone and cerebellum (12.50 [10.28-14.21] vs. 10.53 [8.70-13.63], *P* > 0.999), bone and brainstem (12.50 [10.28-14.21] vs. 10.62 [8.38-12.74], *P* = 0.288), cerebellum and brainstem (10.53 [8.70-13.63] vs. 10.62 [8.38-12.74], *P* > 0.999), vein and CNs (16.00 [14.29-17.81] vs. 15.79 [13.79-18.61], *P* > 0.999).

For session 2, the Wilcoxon matched-pairs test failed to show statistically significant variation between bone and cerebellum (11.24 [9.45-13.29] vs. 9.68 [7.96-12.74], *P* > 0.999), bone and artery (11.24 [9.45-13.29] vs. 13.42 [10.88-15.00], *P* = 0.195), cerebellum and brainstem (11.24 [9.45-13.29] vs. 9.52 [7.48-11.87], *P* > 0.999), artery and CNs (13.42 [10.88-15.00] vs. 14.09 [11.31-17.20], *P* > 0.999), vein and CNs (14.81 [12.94-16.67] vs. 14.09 [11.31-17.20], *P* > 0.999).

For session 3, the Wilcoxon matched-pairs test failed to show statistically significant variation between bone and cerebellum (11.70 [9.62-13.29] vs. 10.53 [7.69-12.44], *P* > 0.999), bone and brainstem (11.70 [9.62-13.29] vs. 10.17 [7.48-11.87], *P* = 0.242), cerebellum and brainstem (10.53 [7.69-12.44] vs. 10.17 [7.48-11.87], *P* > 0.999), vein and CNs (14.67 [13.07-16.28] vs. 14.71 [12.78-17.21], *P* > 0.999).

For session 4, the Wilcoxon matched-pairs test failed to show statistically significant variation between operative space and artery (15.51 [13.68-16.09] vs. 15.07 [12.50-17.02], *P* > 0.999), operative space and vein (15.51 [13.68-16.09] vs. 15.63 [12.21-17.77], *P* > 0.999), operative space and CNs (15.51 [13.68-16.09] vs. 17.14 [14.73-20.00], *P* > 0.999), bone and brainstem (12.90 [11.11-15.24] vs. 12.50 [11.11-13.83], *P* = 0.272), cerebellum and brainstem (11.11 [8.70-13.33] vs. 12.50 [11.11-13.83], *P* > 0.999), artery and vein (15.07 [12.50-17.02] vs. 15.63 [12.21-17.77], *P* > 0.999), vein and CNs (15.63 [12.21-17.77] vs. 17.14 [14.73-20.00], *P* > 0.272).

For session 5, the Wilcoxon matched-pairs test failed to show statistically significant variation between bone and cerebellum (11.65 [9.52-13.46] vs. 10.34 [7.69-12.50], *P* = 0.680), bone and brainstem (11.65 [9.52-13.46] vs. 11.43 [9.56-12.88], *P* > 0.999), bone and artery (11.65 [9.52-13.46] vs. 12.86 [11.11-15.95], *P* = 0.241), cerebellum and brainstem (10.34 [7.69-12.50] vs. 11.43 [9.56-12.88], *P* > 0.999), artery and vein (12.86 [11.11-15.95] vs. 13.84 [11.46-16.67], *P* > 0.999), vein and CNs (13.84 [11.46-16.67] vs. 15.71 [12.38-18.88], *P* = 0.335).

For session 6, the Wilcoxon matched-pairs test failed to show statistically significant variation between bone and cerebellum (11.90 [10.33-13.95] vs. 10.13 [7.69-12.40], *P* = 0.189), bone and brainstem (11.90 [10.33-13.95] vs. 11.48 [10.06-12.50], *P* > 0.999), bone and artery (11.90 [10.33-13.95] vs. 13.73 [11.43-15.38], *P* = 0.067), cerebellum and brainstem (10.13 [7.69-12.40] vs. 11.48 [10.06-12.50], *P* = 0.537), artery and vein (13.73 [11.43-15.38] vs. 14.12 [11.38-17.02], *P* > 0.999), vein and CNs (14.12 [11.38-17.02] vs. 15.60 [13.01-18.43], *P* = 0.306).

For session 7, the Wilcoxon matched-pairs test failed to show statistically significant variation between bone and cerebellum (9.43 [6.25-13.33] vs. 7.69 [6.25-12.50], *P* > 0.999), brainstem and artery (12.91 [11.76-15.69] vs. 16.23 [11.27-20.00], *P* = 0.597), brainstem and vein (12.91 [11.76-15.69] vs. 12.50 [10.53-15.79], *P* > 0.999), artery and vein (16.23 [11.27-20.00] vs. 12.50 [10.53-15.79], *P* = 0.626), artery and CNs (16.23 [11.27-20.00] vs. 16.67 [12.50-20.00], *P* > 0.999).

For session 8, the Wilcoxon matched-pairs test failed to show statistically significant variation between bone and cerebellum (6.91 [5.88-10.97] vs. 7.14 [5.88-11.11], *P* > 0.999), brainstem and artery (11.44 [7.69-12.50] vs. 12.91 [7.69-15.79], *P* = 0.385), brainstem and vein (11.44 [7.69-12.50] vs. 11.33 [6.79-13.22], *P* > 0.999), brainstem and CNs (11.44 [7.69-12.50] vs. 13.81 [8.27-16.67], *P* = 0.093), artery and vein (12.91 [7.69-15.79] vs. 11.33 [6.79-13.22], *P* = 0.276), artery and CNs (12.91 [7.69-15.79] vs. 13.81 [8.27-16.67], *P* > 0.999), vein and CNs (11.33 [6.79-13.22] vs. 13.81 [8.27-16.67], *P* = 0.052).

For session 9, the Wilcoxon matched-pairs test failed to show statistically significant variation between bone and cerebellum (7.02 [6.25-11.87] vs. 7.02 [6.25-11.11], *P* > 0.999), brainstem and artery (11.76 [10.62-13.22] vs. 12.50 [8.19-17.59], *P* > 0.999), brainstem and vein (11.76 [10.62-13.22] vs. 11.23 [9.68-13.22], *P* > 0.999), brainstem and CNs (11.76 [10.62-13.22] vs. 12.70 [11.43-17.21], *P* = 0.053), artery and vein (12.50 [8.19-17.59] vs. 11.23 [9.68-13.22], *P* > 0.999), artery and CNs (12.50 [8.19-17.59] vs. 12.70 [11.43-17.21], *P* > 0.999).

For session 10, the Wilcoxon matched-pairs test failed to show statistically significant variation between bone and artery (13.33 [11.11-15.79] vs. 14.29 [11.76-17.65], *P* > 0.999), bone and vein (13.33 [11.11-15.79] vs. 13.33 [11.76-15.79], *P* > 0.999), cerebellum and brainstem (7.14 [6.25-11.11] vs. 10.53 [6.79-13.12], *P* = 0.068), artery and vein (14.29 [11.76-17.65] vs. 13.33 [11.76-15.79], *P* > 0.999).

For session 11, the Wilcoxon matched-pairs test failed to show statistically significant variation between bone and brainstem (11.11 [7.21-12.50] vs. 7.14 [6.25-11.11], *P* = 0.055), bone and artery (11.11 [7.21-12.50] vs 11.76 [7.85-14.29], *P* > 0.999), bone and vein (11.11 [7.21-12.50] vs. 11.44 [7.21-12.91], *P* > 0.999), cerebellum and brainstem (6.67 [5.88-7.69] vs. 7.14 [6.25-11.11], *P* = 0.265), artery and vein (11.76 [7.85-14.29] vs. 11.44 [7.21-12.91], *P* > 0.999), artery and CNs (11.76 [7.85-14.29] vs. 13.19 [11.76-15.38], *P* = 0.576).

For session 12, the Wilcoxon matched-pairs test failed to show statistically significant variation between bone and brainstem (11.65 [7.85-14.29] vs. 7.85 [7.14-11.43], *P* = 0.054), bone and artery (11.65 [7.85-14.29] vs. 12.66 [10.00-14.81], *P* > 0.999), bone and vein (11.65 [7.85-14.29] vs. 11.76 [7.85-13.33], *P* > 0.999), cerebellum and brainstem (7.14 [5.97-9.22] vs. 7.85 [7.14-11.43], *P* = 0.052), artery and vein (12.66 [10.00-14.81] vs. 11.76 [7.85-13.33], *P* > 0.999).

**Supplementary Table 10. The decision-making in each training session**

| **Sessions** | **ITA, n=62** | | | | **TTA, n=62** | | | | **STA, n=62** | | | |
| --- | --- | --- | --- | --- | --- | --- | --- | --- | --- | --- | --- | --- |
|  | **Optimal** | **Suboptimal** | **Select, but neither optimal nor suboptimal** | **Not select** | **Optimal** | **Suboptimal** | **Select, but neither optimal nor suboptimal** | **Not select** | **Optimal** | **Suboptimal** | **Select, but neither optimal nor suboptimal** | **Not select** |
| Session 1 | 56 (90.32%) | 0 (0.00%) | 1 (1.61%) | 5 (8.06%) | 1 (1.61%) | 40 (64.52%) | 5 (8.06%) | 16 (25.81%) | 0 (0.00%) | 17 (27.42%) | 4 (6.45%) | 41 (66.13%) |
| Session 2 | 0 (0.00%) | 0 (0.00%) | 0 (0.00%) | 62 (100.00%) | 23 (37.10%) | 12 (19.35%) | 27 (43.55%) | 0 (0.00%) | 12 (19.35%) | 23 (37.10%) | 27 (43.55%) | 0 (0.00%) |
| Session 3 | 3 (4.84%) | 0 (0.00%) | 28 (45.16%) | 31 (50.00%) | 11 (17.74%) | 3 (4.84%) | 38 (61.29%) | 10 (16.13%) | 3 (4.84%) | 14 (22.58%) | 24 (38.71%) | 21 (33.87%) |
| Session 4 | 56 (90.32%) | 0 (0.00%) | 1 (1.61%) | 5 (8.06%) | 0 (0.00%) | 41 (66.13%) | 6 (9.68%) | 15 (24.19%) | 0 (0.00%) | 15 (24.19%) | 5 (8.06%) | 42 (67.74%) |
| Session 5 | 0 (0.00%) | 0 (0.00%) | 0 (0.00%) | 62 (100.00%) | 23 (37.10%) | 12 (19.35%) | 27 (43.55%) | 0 (0.00%) | 12 (19.35%) | 23 (37.10%) | 27 (43.55%) | 0 (0.00%) |
| Session 6 | 3 (4.84%) | 0 (0.00%) | 28 (45.16%) | 31 (50.00%) | 11 (17.74%) | 3 (4.84%) | 38 (61.29%) | 10 (16.13%) | 3 (4.84%) | 14 (22.58%) | 24 (38.71%) | 21 (33.87%) |
| Session 7 | 62 (100.00%) | 0 (0.00%) | 0 (0.00%) | 0 (0.00%) | 0 (0.00%) | 62 (100.00%) | 0 (0.00%) | 0 (0.00%) | 0 (0.00%) | 0 (0.00%) | 0 (0.00%) | 62 (100.00%) |
| Session 8 | 0 (0.00%) | 0 (0.00%) | 0 (0.00%) | 62 (100.00%) | 62 (100.00%) | 0 (0.00%) | 0 (0.00%) | 0 (0.00%) | 0 (0.00%) | 62 (100.00%) | 0 (0.00%) | 0 (0.00%) |
| Session 9 | 0 (0.00%) | 12 (19.35%) | 0 (0.00%) | 50 (80.65%) | 0 (0.00%) | 50 (80.65%) | 0 (0.00%) | 12 (19.35%) | 62 (100.00%) | 0 (0.00%) | 0 (0.00%) | 0 (0.00%) |
| Session 10 | 62 (100.00%) | 0 (0.00%) | 0 (0.00%) | 0 (0.00%) | 0 (0.00%) | 62 (100.00%) | 0 (0.00%) | 0 (0.00%) | 0 (0.00%) | 0 (0.00%) | 0 (0.00%) | 62 (100.00%) |
| Session 11 | 0 (0.00%) | 0 (0.00%) | 0 (0.00%) | 62 (100.00%) | 62 (100.00%) | 0 (0.00%) | 0 (0.00%) | 0 (0.00%) | 0 (0.00%) | 62 (100.00%) | 0 (0.00%) | 0 (0.00%) |
| Session 12 | 0 (0.00%) | 12 (19.35%) | 0 (0.00%) | 50 (80.65%) | 0 (0.00%) | 50 (80.65%) | 0 (0.00%) | 12 (19.35%) | 62 (100.00%) | 0 (0.00%) | 0 (0.00%) | 0 (0.00%) |

ITA = inferior-tubercle approach; n = the number of subjects; STA = superior-tubercle approach; TTA = trans-tubercle approach.

Percentages do not add up to 100% due to rounding.
